# Perforin-2 enhances antigen-specific CTL immune response by promoting cross presentation

**DOI:** 10.1038/s41419-026-08705-1

**Published:** 2026-04-09

**Authors:** Zhi-kai Zha, Cheng-jie Deng, Ling-jun Shen, Ling-zhen Liu, Yun-xiao Huang, Yan-hong Li, Li Lv, Ke Zhang, Lin-shuang Chen, Fei-er Chen, Sheng-an Li

**Affiliations:** 1 Yunnan Provincial Key Laboratory of Public Health and Bio-safety & Department of Pathogen Biology and Immunology, Faculty of Basic Medical Science, Kunming Medical University, Kunming, China; 2 The Center of Experimental Teaching, Faculty of Basic Medical Science, Kunming Medical University, Kunming, China; 3 Department of Tuberculosis, Yunnan Clinical Medical Center for Infectious Diseases, the Third People’s Hospital of Kunming (The Sixth Affiliated Hospital of Dali University), Kunming, China; 4 Key Laboratory of Sepsis and Brain Regulation in Universities of Yunnan Province, Faculty of Basic Medical Science, Kunming Medical University, Kunming, China

**Keywords:** Antigen-presenting cells, Tumour immunology

## Abstract

Antigen cross-presentation is essential for initiating CD8^**+**^ cytotoxic T lymphocyte (CTL) response. Perforin-2 (P2), a pore-forming protein constitutively expressed in dendritic cells (DCs), has been implicated in endocytic cargo escape, but its role in cross-presentation remains poorly defined. Here, we show that loss of P2 markedly impairs DC-mediated cross-presentation of both soluble and particulate antigens, leading to weakened antigen-specific CD8^**+**^ T cell responses. Additionally, *P2*^*-/-*^ mice exhibited defective endogenous CTL responses and diminished anti-tumor immunity in melanoma models. Mechanistically, oligomerization of plasma membrane P2 promotes antigen uptake via membrane-repair-mediated macropinocytosis. In parallel, P2 limits excessive endosomal acidification, preserving antigens for efficient loading onto MHC class I molecules. These findings identify P2 as a key regulator that coordinates antigen uptake and processing to support effective cross-presentation and CD8^+^ T cell immunity.

## Introduction

The presentation of exogenous antigens by dendritic cells (DCs) via MHC I molecules is termed cross-presentation, which is crucial for the initiation of CD8^**+**^ cytotoxic T lymphocyte (CTL) responses against tumors and intracellular pathogens [1–3]. Two major pathways mediate antigen cross-presentation in DCs: the cytosolic pathway, which involves exogenous antigen translocation from endosomes or phagosomes into the cytosol, and the vacuolar pathway, which does not [2]. Both antigen uptake and intracellular processing by DCs are pivotal for efficient cross-presentation and are tightly regulated. Apart from acquiring antigens through phagocytosis [4], receptor-mediated antigen uptake, such as via the mannose receptor, DEC-205, and DNGR-1 has been well characterized [5, 6]. The effectiveness of cross-presentation also depends on the intracellular processing of antigens, particularly the levels of endolysosomal pH and protease activity [7, 8]. For example, Sec61 is recruited into endosomes to facilitate antigen transport from endosomes to the cytosol for cross-presentation [9]. Given the complexity of cross-presentation, exploring new regulators is helpful for further understanding this process.

Pore forming proteins (PFPs) are a unique class of membrane-damaging proteins, which form oligomerization pores in lipid bilayer membrane [10]. These proteins are produced in various organisms for diverse purposes. In pathogens, they may facilitate host invasion, whereas in the host, PFPs serve as potent weapons against pathogens and tumor cells either through direct killing or by modulating the immune response [11]. For example, perforin-1 and gasdermin B participate in immune responses through different mechanisms: the former is a key effector molecule for cytotoxic lymphocyte lysis of target cells, and the latter enhances anti-tumor immunity by triggering target cell pyroptosis [12, 13]. Emerging evidence suggests that different classes of PFPs can promote cross-presentation-mediated CTL responses. Sticholysin II, a PFP derived from the Sea anemone *Stichodactyla helianthus*, effectively facilitates OVA-specific CD8^**+**^ T cells immune responses [14], while the apolipoprotein APOL7C promotes the delivery of exogenous antigens into the cytosol to support cross-presentation [15]. Notably, our previous studies on the amphibian PFP βγ-CAT have shown that the protein strengthens antigen uptake by DCs and actively neutralizes the acidification of intracellular endolysosome to enhance antigen cross-presentation, leading to a robust CTL response [16, 17]. With these in mind, the role of host endogenous PFPs in cross-presentation may be interesting and promising.

Perforin-2 (P2), a PFP encoded by the *perforin-2* gene (also known as *MPEG1*), is a highly conserved protein in vertebrate and belongs to the membrane attack complex/perforin protein (MACPF) superfamily [18, 19]. Originally identified in macrophages, P2 is also expressed in DCs and neutrophils [19]. P2 forms ~100 Å pores in bacterial membranes, facilitating microbial killing by allowing the entry of proteases and antimicrobial agents [20, 21]. Beyond microbial defense, P2 may provide channels for intracellular release of IL-33 on the plasma membrane and promote anti-helminth immunity [22]. Strikingly, a recent investigation has indicated that P2 is not required for macrophages or DCs to directly kill bacteria, but rather may be involved in adaptive immunity, such as antigen presentation [23]. Although this notion seems to be partially supported by another study confirming that P2 promotes the escape of endocytic contents [24], its role in cross-presentation and CTL responses remains poorly defined.

To address this gap, we employed P2 knockout (*P2*^*-/-*^) mice and generated bone marrow-derived DCs (BMDCs) to investigate the role of P2 in cellular immunity. We demonstrate that P2 is essential for cross-presentation and for priming endogenous antigen-specific CTL responses to both soluble and cell-associated antigens. *P2*^*-/-*^ mice fail to adequately prime endogenous CD8^**+**^ cytotoxic T cells to suppress tumor growth. P2 promotes antigen uptake and modulates intracellular antigen processing in immature DCs. Our results highlight the critical role of P2 in cross-presentation and CTL immunity.

## Results

### P2 deficiency impairs DC-mediated cross-priming of CD8^+^ T cells

P2 is predominantly expressed in antigen-presenting cells (APCs) such as DCs, macrophages [20]. To assess its function, we generated P2 knockout mice and derived *P2*^*−/−*^ BMDCs using GM-CSF cultures [25, 26]. Flow cytometry showed that P2 deficiency did not affect BMDC differentiation, as the frequencies of the CD24^+^ (CD11c^+^MHC II^+^CD11b^int^) and SIRPα^+^ (CD11c^+^MHC II^+^CD11b^high^) subsets within the CD11c^+^MHC II^+^ population were comparable between genotypes (Fig. S1A–C). Western blot analysis confirmed robust P2 expression in *P2*^*+/+*^ BMDCs and its complete absence in *P2*^*-/-*^ cells (Fig. S1D). Given that BMDC maturation is critical for antigen presentation [6], we next examined co-stimulatory molecule expression. LPS stimulation induced comparable upregulation of CD40, CD80, and CD86 in BMDCs from both genotypes (Fig. S1E). Moreover, surface abundance of MHC I and MHC II was comparable between genotypes (Fig. S1F, G).

**Fig. 1:**
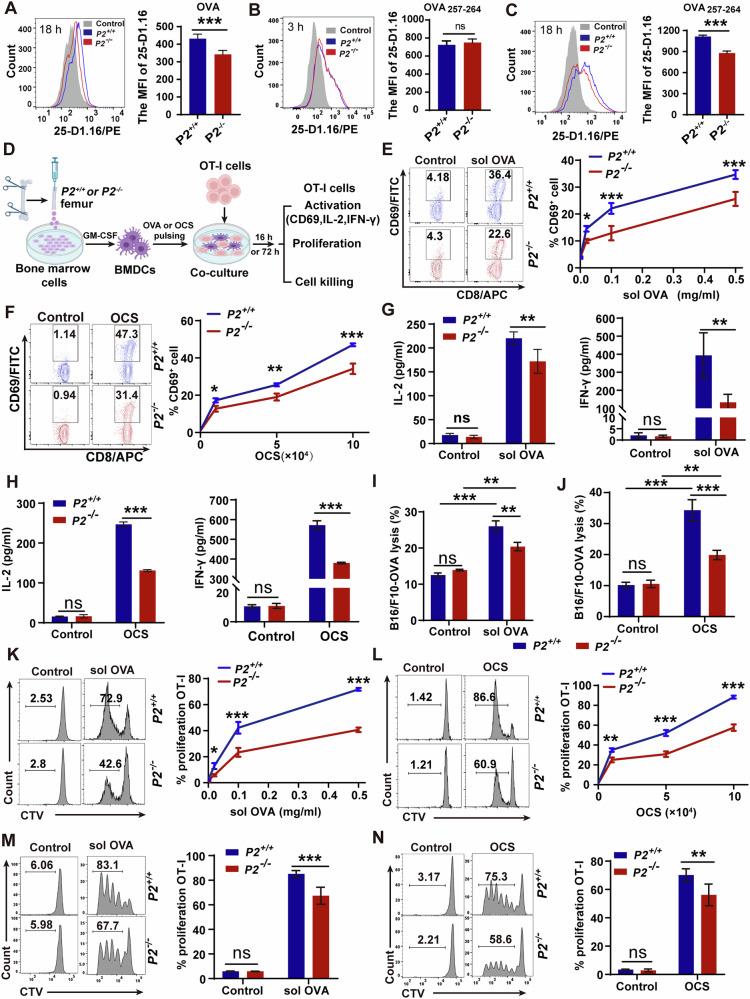
P2 deficiency impairs DC-mediated cross-priming of CD8^+^ T cells. **A** Flow cytometry histograms showing MFI of 25-D1.16 staining on *P2*^*+/+*^ and *P2*^*-/-*^ BMDCs after 18 h incubation with 2 mg/ml OVA protein. **B**, **C** Flow histograms showing MFI of 25-D1.16 on BMDCs incubated with 5 μg/ml OVA_**257-264**_ peptide for 3 h (**B**) or 18 h (**C**). **D** Schematic of the co-culture experiment to assess BMDC-mediated OT-I T cell activation. **E**, **F** Percentage of CD8^+^CD69^+^ T cells after co-culture of OT-I cells with BMDCs pre-treated with indicated amounts of soluble OVA (**E**) or OCSs (**F**). **G**, **H** IL-2 and IFN-γ concentrations (ELISA) in supernatants from OT-I cells co-cultured for 16 h with BMDCs pulsed with 0.5 mg/ml soluble OVA (**G**) or 1×10⁵ OCSs (**H**). **I**, **J** Specific killing of B16/F10-OVA target cells by OT-I T cells primed by BMDCs treated with 0.5 mg/ml soluble OVA (**I**) or 1×10⁵ OCSs (**J**). **K**, **L** Proliferation of OT-I CD8^+^ T cells (assessed by CTV dilution) after co-culture with BMDCs treated with soluble OVA (**K**) or OCSs (**L**); values indicate percentage of divided cells. **M**, **N** In vivo proliferation of adoptively transferred CTV-labeled OT-I cells in *P2*^*+/+*^ and *P2*^*-/-*^ mice (*n* = 6) injected with soluble OVA (**M**) or OCSs (**N**); values indicate percentage of divided splenic OT-I T cells (pre-gated on PI^-^CD3^+^CD8^+^TCR Vα2^+^ cells). Data represent one of three independent experiments, and shown as mean ± SD; ns, not significant (*P* > 0.05); *P < 0.05, **P < 0.01 and ***P < 0.001 by unpaired two-tailed Student’s *t* test (**A–C**) and two-way ANOVA with Šídák’s multiple comparisons test (**E–N**).

To directly evaluate the functional role of P2 in antigen cross-presentation, we pulsed BMDCs with full-length OVA protein for 18 h and quantified surface MHC I-OVA_**257-264**_ complexes using the 25-D1.16 antibody. Notably, P2-deficient BMDCs exhibited significantly reduced 25-D1.16 signal compared to wild-type BMDCs (Fig. 1A), revealing a specific defect in cross-presentation. To distinguish between impaired peptide loading and upstream antigen processing defects, we directly pulsed BMDCs with the OVA_**257-264**_ peptide. Short-term incubation (3 h) showed no significant differences in 25-D1.16 signal between P2-deficient and wild-type BMDCs (Fig. 1B), indicating P2 is not involved in the direct binding of OVA_**257–264**_ peptide to MHC I on the cell surface. However, after prolonged exposure (18 h), P2-deficient BMDCs displayed markedly diminished signal compared to their *P2*^*+/+*^ counterparts (Fig. 1C), indicating that P2 acts upstream of peptide loading during antigen cross-presentation.

DCs initiate the CD8^+^ T cell response by presenting MHC I-antigen peptide complexes to naive CD8^+^ T cells [27]. However, enhanced antigen cross-presentation does not invariably augment CD8^+^ T cells cross-priming [28]. Given P2’s role in antigen cross-presentation (Fig. 1A–C), we investigated whether P2 deficiency impairs DC-mediated cross-priming for both soluble and particulate antigens. *P2*^*-/-*^ or *P2*^*+/+*^ BMDCs were pulsed with soluble OVA or OVA-coated irradiated splenocytes (OCSs) and co-cultured with naive OT-I CD8^+^ T cells (Fig. 1D). To exclude contributions from endogenous APCs, we rendered OCSs non-stimulatory by UV irradiation (Fig. S2A). *P2*^*-/-*^ BMDCs exhibited significantly diminished cross-priming of naive OT-I CD8^**+**^ T cells, evidenced by a lower proportion of T cells expressing the CD69 molecule (Fig. 1E, F), and markedly reduced production of IFN-γ and IL-2 in the DC-T cells co-culture supernatants (Fig. 1G, H). Notably, OVA stimulation alone did not induce detectable production of these cytokines in BMDCs supernatant (Fig. S1H, I), indicating that the reduced cytokine levels observed in co-cultures reflect impaired T-cell activation. Furthermore, when BMDCs were pulsed with either soluble OVA or OCSs, *P2*^*-/-*^ BMDCs primed OT-I T cells with markedly reduced cytotoxic activity toward OVA-bearing target cells compared with *P2*^*+/+*^ BMDCs (Fig. 1I, J; Fig. S2B). Consistent with this defect, CTV dilution assays confirmed impaired proliferation of these OT-I T cells in co-cultures with *P2*^*-/-*^ BMDCs (Fig. 1K, L). To validate these findings in vivo, CTV-labeled OT-I cells were adoptively transferred into *P2*^*-/-*^ and *P2*^*+/+*^ mice, followed by intravenous injection of soluble OVA or OCSs. Proliferation of OT-I cells in the spleen was markedly impaired in *P2*^*-/-*^ mice, with the overall number of expanded OT-I cells reduced by 10-20% compared to *P2*^*+/+*^ mice (Fig. 1M, N; Fig. S2C, D). Collectively, these results suggest that P2 is essential for cross-priming of CD8^+^ T cells by APCs against both soluble and particulate antigens.

Given the cellular heterogeneity of GM-CSF-derived BMDCs [25], we directly assessed P2’s function in cross-priming by primary conventional DCs from the spleen. Flow-sorted cDC1 (CD11c^+^B220^-^XCR1^+^) and cDC2 (CD11c^+^B220^-^SIRPα^+^) cells from *P2*^*-/-*^ or *P2*^*+/+*^ mice (Fig. S2E–G) were first analyzed by immunoblot for P2 expression. P2 was abundantly expressed in *P2*^*+/+*^ cDC1 but barely detectable in cDC2, and was completely absent in both subsets from *P2*^*-/-*^ mice (Fig. S2H). Functionally, P2 deficiency severely impaired the capacity of cDC1 to activate OT-I T cells (Fig. S2I), whereas did not alter the ability of cDC2 to prime OT-I cells (Fig. S2J), consistent with the expression pattern of P2. Furthermore, although P2 protein was absent in *P2*^*-/-*^ mouse bone marrow-derived macrophages (BMDMs) (Fig. S2K), it exhibited similar cross-activation ability of OT-I cells in both genotypes in vitro (Fig. S2L). These findings corroborate our observations in BMDCs, indicating that P2 intrinsically regulates the cross-priming capacity in DCs.

### P2 promotes endogenous CD8^+^ T cell responses

**Fig. 2:**
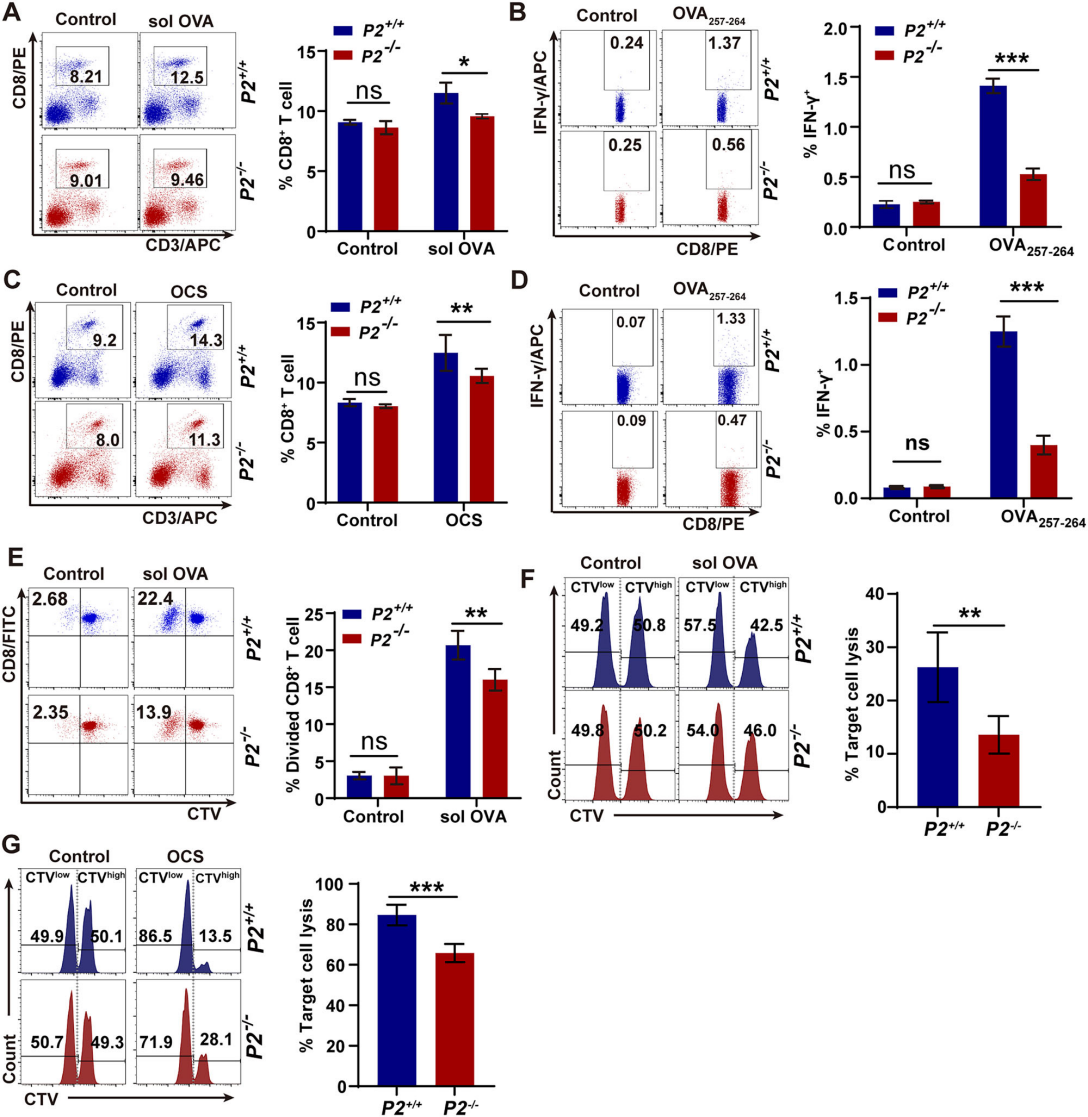
P2 promotes endogenous CD8^+^ T cell responses. **A**, **B** Flow cytometric analysis of splenic CD8^+^ T cells in *P2*^*-/-*^ and *P2*^*+/+*^ mice immunized with soluble OVA showing total CD3^+^CD8^+^ T cell populations (**A**) and IFN-γ-producing CD8^+^ T cells after OVA_**257-264**_ peptide restimulation (**B**). **C**, **D** Parallel analysis of splenic CD8^+^ T cells in OCSs-immunized mice demonstrating total CD3^+^CD8^+^ T cells (**C**) and OVA-specific IFN-γ-producing CD8^+^ T cells after OVA_**257-264**_ peptide restimulation (**D**). **E** Proliferation of splenic CD8^+^ T cells from immunized mice after in vitro OVA re-stimulation. **F**, **G** Endogenous CTL responses in mice primed with soluble OVA (**F**) or OCSs (**G**) showing specific lysis of target cells. Data represent one of three independent experiments, and shown as mean ± SD; ns, not significant (*P* > 0.05); *P < 0.05, **P < 0.01, ***P < 0.001 by two-way ANOVA with Šídák’s multiple comparisons test (**A–E**) and unpaired two-tailed Student’s *t* test (**F**, **G**).

Although P2 enhances cross-presentation of soluble or particulate OVA antigens to OVA-specific OT-I cells (Fig. 1), its role in endogenous CD8^+^ T cell responses remained unknown. Given unaltered the frequencies of immune cells in P2 deficiency mice (Fig. S3), we next investigated the role of P2 in endogenous CD8^+^ T cells responses against sol OVA or OCSs antigens. Flow cytometry revealed significantly reduced total CD8^+^ T cells abundance in spleen of *P2*^*-/-*^ mice versus *P2*^*+/+*^ mice under both types antigen challenge (Fig. 2A, C). Consistently, restimulation with OVA_**257-264**_ peptide showed 2-fold lower IFN-γ^+^CD8^+^ T cells in *P2*^*-/-*^ mice (Fig. 2B, D), indicating lower OVA-specific CD8^+^ T cell responses in P2 deficient mice. Additionally, similar defects occurred in CD4^+^ T cell responses of *P2*^*-/-*^ mice following immunization with both antigens (Fig. S4A–E).

To functionally validate this defect, splenocytes from OCS-primed mice were CTV-labeled and restimulated with soluble OVA in vitro. *P2*^*-/-*^ mice exhibited significantly reduced OVA-specific CD8^+^ T cell expansion (Fig. 2E). To evaluate the impact of P2-dependent cross-presentation on cytotoxic T lymphocyte (CTL) effector function in vivo, we immunized *P2*^*+/+*^ and *P2*^*-/-*^ mice with the soluble OVA or OCS. After T cell priming, in vivo CTL activity was quantified. Compared to *P2*^*+/+*^ mice, *P2*^*-/-*^ mice showed significantly reduced lysis efficiency of target cells (Fig. 2F, G; Fig. S4F). As P2 was undetectable in splenic T cells under antigen-immunized and non-immunized condition (Fig. S4G), impaired CTL activity unequivocally reflects defective cross-presentation rather than T cell dysfunction. Collectively, P2 is essential for generating endogenous CD8^+^ T cell responses against diverse antigens.

### P2 deficiency weakens anti-tumor T cell immunity

**Fig. 3:**
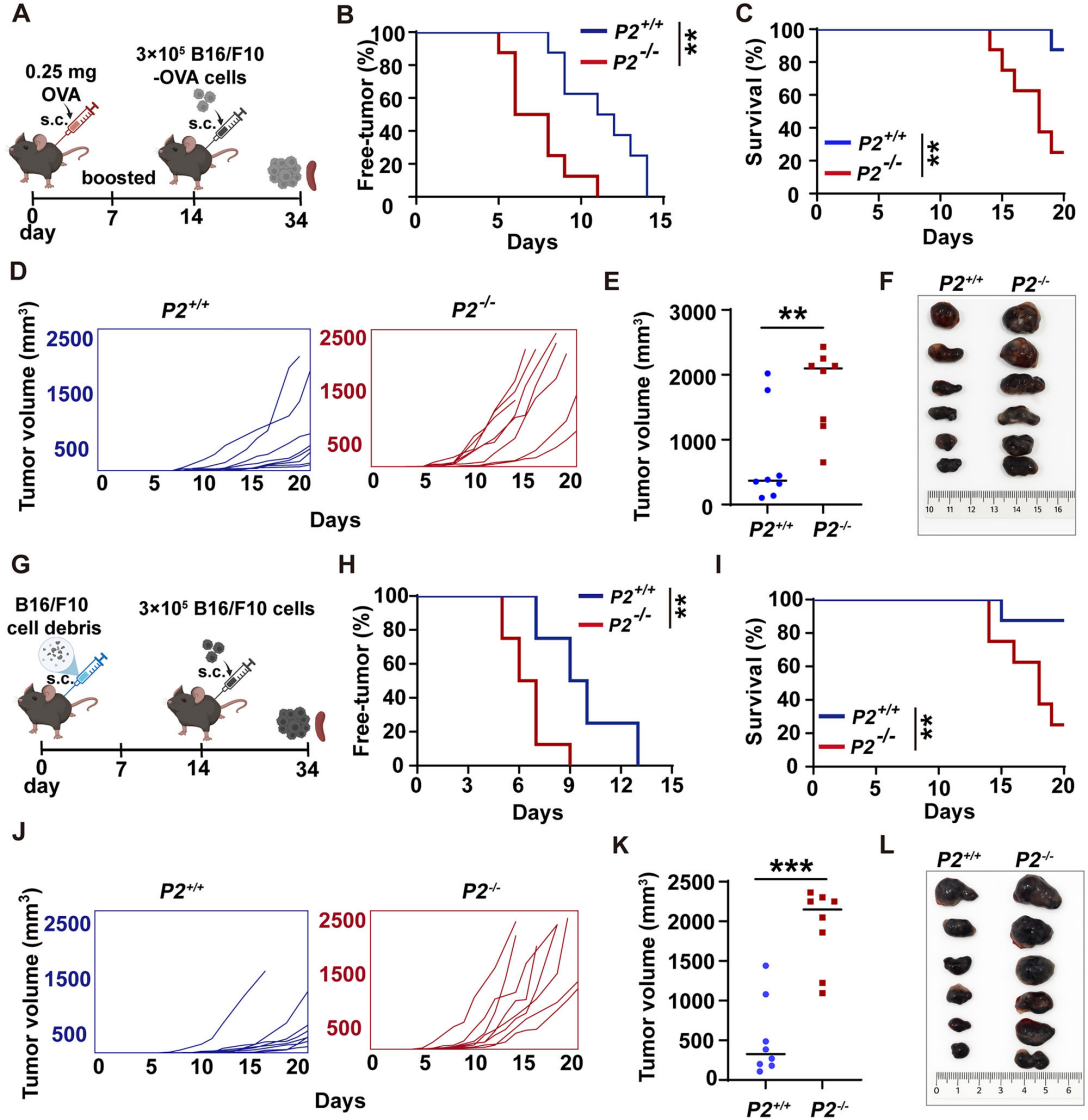
P2 deficiency aggravates tumor progression and curtails survival in mice. **A** Schematic of the B16/F10-OVA tumor model. **B**, **C** Tumor-free (**B**) and survival curves (**C**) of *P2*^*-/-*^ and *P2*^*+/+*^ mice (*n* = 8) challenged with B16/F10-OVA cells. **D**, **E** Tumor growth curves (**D**) and final tumor volumes in B16/F10-OVA-bearing mice (**E**). **F** Representative tumor images on day 19 post-inoculation. **G** Schematic of the B16/F10 tumor model. **H**, **I** Tumor-free (**H**) and survival curves (**I**) in B16/F10-challenged mice. **J**, **K** Tumor growth curves (**J**) and final volumes (**K**) in B16/F10-bearing mice. **L** Representative tumor images on day 15 post-inoculation. Statistically significant differences were estimated by unpaired two-tailed Student’s *t* test (**E**, **K**) and the log-rank test (**B**, **C**, **H**, **I**). All results are representative of two independent experiments, and shown as mean ± SD; **P < 0.01, ***P < 0.001.

DCs orchestrate anti-tumor immunity by cross-presenting tumor antigens to activate and expand tumor-specific CD8^+^ T cells [29]. Given P2’s important role in antigen cross-presentation (Figs. 1 and 2), we investigated its impact on anti-tumor immunity. We first examined the clearance of highly immunogenic OVA-expressing melanoma (B16/F10-OVA) (Fig. 3A). B16/F10-OVA tumors emerged earlier (Fig. 3B), and caused significantly shortened survival in *P2*^*-/-*^ mice versus *P2*^*+/+*^ mice (Fig. 3C). The overall tumor burden was markedly enhanced in *P2*^*-/-*^ mice compared to their *P2*^*+/+*^ counterparts, including accelerated growth kinetics and increased final tumor volume (Fig. 3D–F). To determine whether P2’s function extends beyond model antigens, we challenged mice with wild-type B16/F10 melanoma (Fig. 3G), which expresses a repertoire of endogenously expressed tumor antigens such as gp100 [30]. Consistent with the B16/F10-OVA tumor model, *P2*^*+/+*^ mice showed delayed onset of B16/F10 tumors (Fig. 3H), higher survival rates (Fig. 3I), and restrained tumor progression, reflected by slower growth and reduced final tumor size (Fig. 3J–L), compared to P2-deficient mice.

**Fig. 4:**
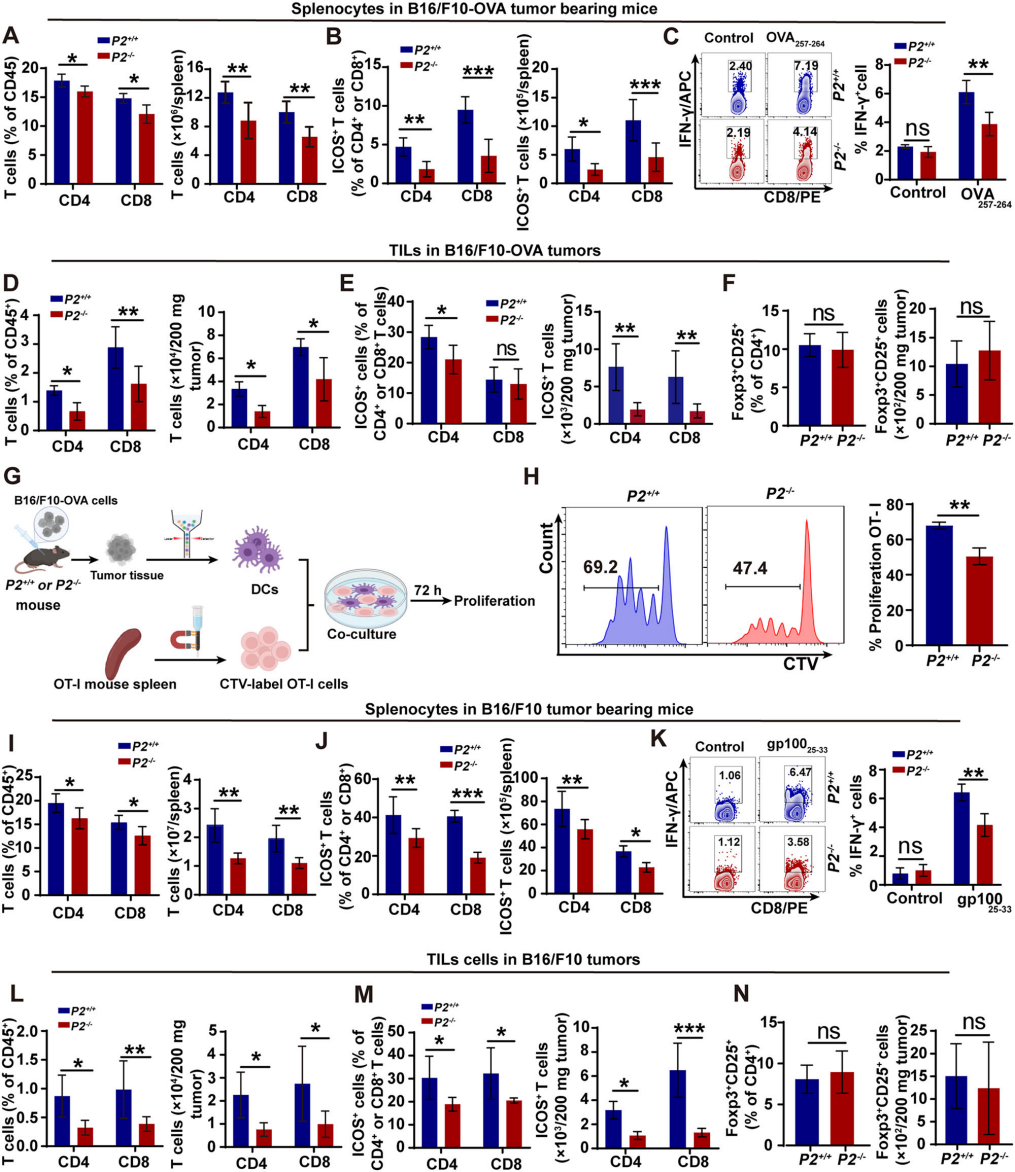
P2 deficiency impairs systemic and intratumoral T-cell responses, leading to compromised antitumor immunity. **A–C** Flow cytometric analysis of splenocytes from B16/F10-OVA tumor-bearing mice. **A** Frequencies and numbers of CD8^+^ and CD4^+^ T cells among CD45^+^ cells. **B** Frequencies and absolute numbers of ICOS^+^CD8^+^ and COS^+^CD4^+^ T cells. **C** IFN-γ production by CD8^+^ T cells after ex vivo stimulation with 10 μg/ml OVA_**257-264**_ peptide. **D–F** Tumor-infiltrating lymphocytes (TILs) in B16/F10-OVA tumors. **D** Frequencies and numbers of CD8^+^ and CD4^+^ T cells. **E** Frequencies and numbers of ICOS^+^CD8^+^ and ICOS^+^CD8^+^ T cells. **F** Frequencies and numbers of tumor-infiltrating Treg cells (Foxp3^+^CD25^+^CD4^+^). **G, H** Ex vivo DC-T cell co-culture assay. **G** Schematic of experimental design: tumor-derived DCs from *P2*^*+/+*^ or *P2*^*-/-*^ tumor-bearing mice were sorted and co-cultured with CTV-labeled OT-I T cells. **H** Representative proliferation histograms and quantification of OT-I proliferation after 72 h. **I–K** Flow cytometric analysis of splenocytes from B16/F10 tumor-bearing mice. **I** Frequencies and numbers of CD8^+^ and CD4^+^ T cells. **J** Frequencies and absolute numbers of ICOS^+^CD8^+^ and COS^+^CD4^+^ T cells. **K** IFN-γ production by CD8^+^ T cells after ex vivo stimulation with 10 μg/ml gp100_**25-33**_ peptide. **L–N** TILs in B16/F10 tumors. **L** Frequencies and numbers of CD8^+^ and CD4^+^ T cells. **M** Frequencies and numbers of ICOS^+^CD8^+^ and COS^+^CD4^+^ T cells. **N** Frequencies and numbers of Foxp3^+^CD25^+^CD4^+^ Treg cells. Statistically significant differences were estimated by two-way ANOVA with Šídák’s multiple comparisons test (**A–E** and **I–M**) and unpaired two-tailed Student’s *t* test (**F**, **H**, **N**). All results are representative of two independent experiments, and shown as mean ± SD; *n* = 6 mice per group; ns, not significant (*P* > 0.05); *P < 0.05, **P < 0.01, ***P < 0.001.

Our previous findings demonstrated that P2 deficiency dampens endogenous CD8^+^ T cell responses (Fig. 2), which are the major cytotoxic immune cells controlling tumor progression. To determine whether P2 deficiency affects anti-tumor T-cell immunity, we performed flow cytometry analysis, which revealed a marked reduction in both the frequency and absolute number of CD8^+^ and CD4^+^ T cells in the spleens of *P2*^*-/-*^ mice bearing B16/F10-OVA tumors (Fig. 4A; Fig. S5A). Of note, PD-1^+^ T cells were comparable between genotypes (Fig. S5B), but the proportion and number of ICOS^+^CD8^+^ and ICOS^+^CD4^+^ T cells were significantly decreased in *P2*^*-/-*^ mice (Fig. 4B; Fig. S5C), indicating impaired systemic T-cell activation. Functionally, upon ex vivo restimulation with OVA_**257-264**_ peptide, splenic CD8^+^ T cells from *P2*^*-/-*^ mice produced markedly less IFN-γ (Fig. 4C), demonstrating weakened antigen-specific cytotoxic T-cell responses.

Additionally, analysis of tumor-infiltrating lymphocytes revealed a reduced frequency of both CD8^+^ and CD4^+^ T cells among CD45^+^ immune cells in tumors from *P2*^*-/-*^ mice (Fig. 4D; Fig. S5D). Moreover, the abundance of ICOS^+^ T cells was lower in *P2*^*-/-*^ tumors (Fig. 4E; Fig. S5E), indicating attenuated local activation of effector T cells. Although PD-1^+^ T cells were enriched in *P2*^*-/-*^ tumors (Fig. S5F), both the frequency and absolute number of tumor-infiltrating Treg cells were comparable between genotypes (Fig. 4F; Fig. S5G), indicating that the impaired tumor control in P2-deficient mice is unlikely to be driven by increased Treg accumulation. Supporting this notion, ex vivo co-culture assays demonstrated that *P2*^*-/-*^ tumor-derived DCs elicited significantly less OT-I T-cell proliferation than their *P2*^*+/+*^ counterparts (Fig. 4G, H), consistent with defective antigen cross-priming capacity.

To further determine whether this defect extends beyond OVA-specific immune responses, we next evaluated the parental B16/F10 tumor model, which does not express OVA. Similar to the findings in the B16/F10-OVA model, the frequency and number of CD8^+^ T cells were markedly reduced in *P2*^*-/-*^ spleens (Fig. 4I; Fig. S6A). Again, while PD-1^+^ T-cell levels were unchanged (Fig. S6B), both ICOS^+^CD8^+^ and ICOS^+^CD4^+^ T cells were significantly decreased in *P2*^*-/-*^ mice (Fig. 4J; Fig. S6C), indicating that P2 deficiency compromises global T-cell activation. Consistently, ex vivo restimulation of splenocytes with the tumor-associated gp100_**25-33**_ peptide revealed markedly reduced IFN-γ production by CD8^+^ T cells from *P2*^*-/-*^ mice (Fig. 4K).

Consistent with these defects, the numbers of CD8^+^ and CD4^+^ T cells in tumors from *P2*^*-/-*^ mice were significantly lower than those in *P2*^*+/+*^ mice (Fig. 4L; Fig. S6D), accompanied by reduced ICOS^+^ T-cell activation (Fig. 4M; Fig. S6E). Although PD-1^+^ T cells were relatively enriched in *P2*^*-/-*^ tumors (Fig. S6F), Treg frequencies and absolute numbers remained comparable between genotypes (Fig. 4N; Fig. S6G), again arguing against enhanced Treg-mediated immunosuppression as a major contributor. Collectively, these findings suggest that P2 deficiency impairs anti-tumor T cells immune.

### P2 promotes antigen internalization by DCs

**Fig. 5:**
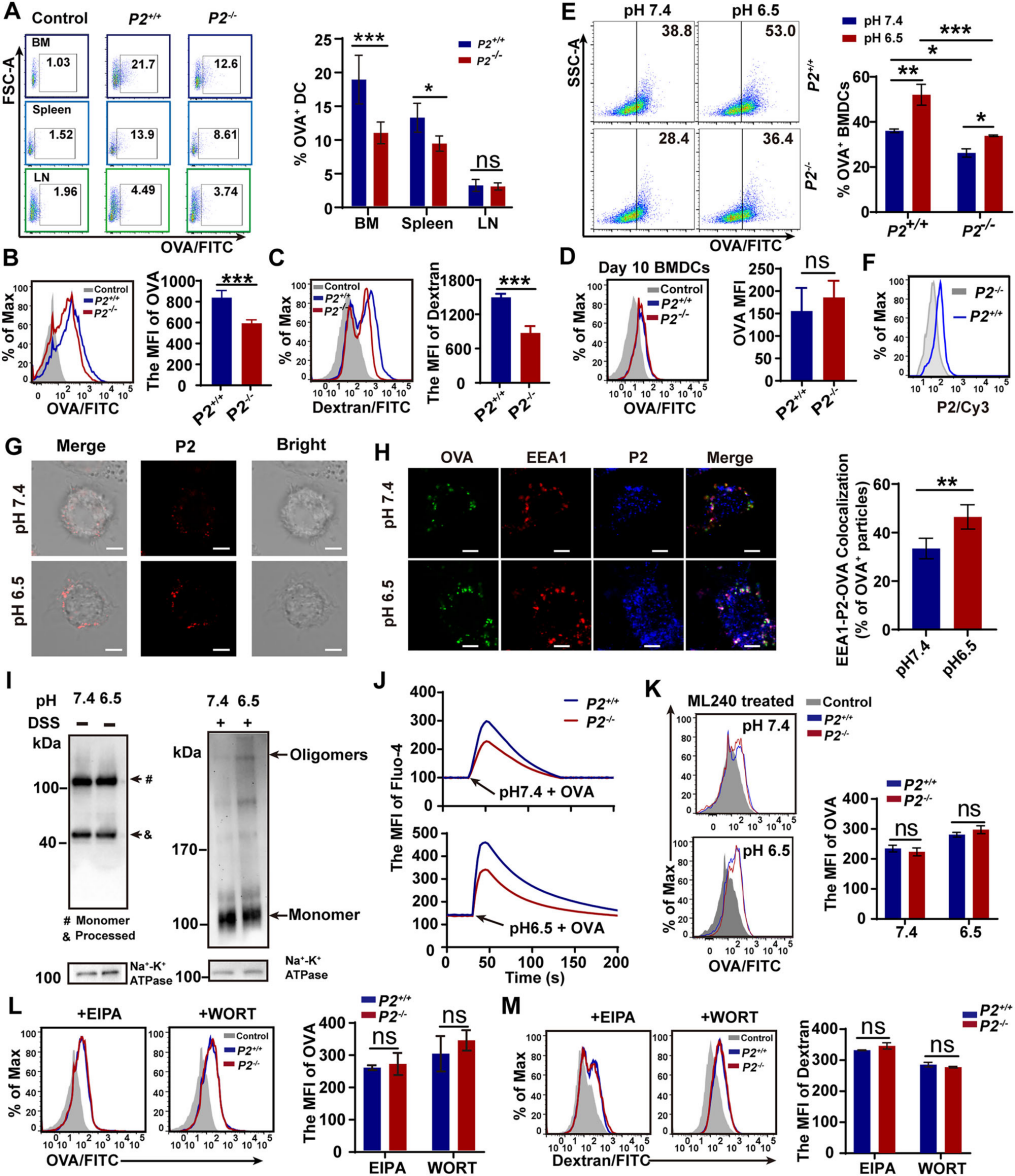
P2 promotes antigen internalization by immature DCs. **A** FITC-OVA uptake by CD11c^+^MHCII^+^ DCs in bone marrow (BM), spleen and lymph nodes of *P2*^*+/+*^ or *P2*^*-/-*^ mice; **B**, **C** Flow cytometric analysis of FITC-OVA (**B**) and FITC-dextran (**C**) uptake by BMDCs showing MFI values. **D** Flow cytometric analysis of FITC-OVA uptake by day-10 BMDCs showing MFI values. **E** pH-dependent OVA internalization in BMDCs at pH 7.4 and 6.5. **F** Surface expression of P2 on non-permeabilized BMDCs by flow cytometry. **G** pH-dependent membrane distribution of P2 detected by immunofluorescence. Scale bar, 5 μm. **H** Co-localization of OVA with P2 and early endosomal marker EEA1. Scale bar, 5 μm. **I** Detection of P2 oligomerization on cell membrane at different pH conditions by immunoblotting. **J** Intracellular Ca²^+^ flux in BMDCs stimulated with OVA at different pH values. **K** MFI of OVA internalization by *P2*^*+/+*^ or *P2*^*-/-*^ BMDCs following ML240 treatment. **L, M** MFI of OVA (**L**) and dextran (**M**) internalization by *P2*^*+/+*^ or *P2*^*-/-*^ BMDCs following treatment with EIPA and wortmannin (WORT). All results are respective three independent experiments, and data shown as mean ± SD; ns, not significant (*P* > 0.05); *P < 0.05, **P < 0.01, ***P < 0.001 by the two-way ANOVA with Šídák’s multiple comparisons test (**A**, **E**, **K–M**) and the unpaired two-tailed Student’s *t* test (**B–D**, **H**).

Given the observed defect in cross-presentation in P2-deficient DCs (Fig. 1, Fig. 2 and Fig. 4G, H), we next examined OVA internalization, the critical first step in the antigen presentation cascade. In vivo uptake assays revealed markedly reduced antigen internalization by DCs from *P2*^*-/-*^ mice, particularly in the bone marrow and spleen (Fig. 5A; Fig. S7A). Consistent with this, *P2*^*-/-*^ BMDCs exhibited significantly reduced uptake of both OVA and the fluid-phase tracer dextran compared to *P2*^*+/+*^ controls (Fig. 5B, C; Fig. S7B, C). This defect was most pronounced in immature BMDCs and was no longer apparent after they reached a mature state (Fig. 5D; Fig. S7D–F), suggesting that P2 primarily facilitates antigen internalization in immature DCs, which rely heavily on macropinocytosis [31]. Given that P2 activity is pH sensitive and that tumor environments are often mildly acidic [32, 33], we further tested OVA uptake under pH 7.4 or 6.5. Acidic conditions markedly enhanced uptake in *P2*^*+/+*^ BMDCs (from 38.8 to 53%) but only modestly in *P2*^*-/-*^ cells (from 28.4 to 36.4%), with comparable viability across groups (Fig. 5E; Fig. S7G), indicating that an acidic environment further potentiates P2-dependent macropinocytosis.

To further elucidate the mechanism by which P2 facilitates antigen uptake, we first examined its cellular distribution. Flow cytometry and immunofluorescence confirmed the presence of P2 on the plasma membrane of *P2*^*+/+*^ BMDCs, and a more clustered membrane pattern was observed under pH 6.5 conditions (Fig. 5F, G). Given the importance of endosomal trafficking during antigen uptake, we next examined the spatial relationship among P2, OVA, and the early endosomal marker EEA1. Confocal microscopy revealed prominent colocalization of these three molecules (Fig. S7H). Under physiological pH, P2 predominantly associated with EEA1^+^ early endosomes containing OVA (Fig. 5H, top), and the proportion of such triple-positive vesicles increased markedly under mildly acidic conditions (pH 6.5; Fig. 5H, bottom). These results indicate that plasma membrane-associated P2 involved in antigen internalization.

For pore-forming proteins of the MACPF/CDC family, oligomerization at the plasma membrane typically induces Ca^2+^ influx, a well-established indicator of membrane damage that activates repair responses [34, 35]. We next examined whether it undergoes similar oligomerization at the plasma membrane. Chemical crosslinking followed by immunoblotting revealed discrete P2 multimers on *P2*^*+/+*^ BMDC membranes, with their abundance markedly increased under low-pH conditions (Fig. 5I), indicating low-pH-induced oligomerization. To determine whether this oligomerization is functionally associated with Ca^2+^ signaling, we measured intracellular Ca^2+^ dynamics. Following OVA stimulation, *P2*^*+/+*^ BMDCs exhibited stronger Ca^2+^ influx than *P2*^*-/-*^ cells at pH 7.4, and this difference became more pronounced under acidic conditions (pH 6.5) (Fig. 5J; Fig. S7I, J).

Having established links between P2 pores, Ca^2+^ influx, and antigen endocytosis, we sought to identify the repair pathway involved. The ESCRTs complex is a critical component of one major plasma membrane repair [36]. Inhibition of the ESCRTs-III-associated ATPases with ML240 abolished the enhanced OVA uptake observed in *P2*^*+/+*^ BMDCs under both physiological and acidic conditions (Fig. 5K). We further employed the macropinocytosis inhibitors EIPA and wortmannin, both of which markedly inhibited P2-enhanced antigen uptake (Fig. 5L, M), indicating that the P2-triggered repair response is coupled to macropinocytic antigen internalization.

Together, these findings reveal a P2 oligomerization-induced membrane repair that promotes macropinocytic antigen uptake.

### P2 inhibits excessive antigen degradation

**Fig. 6:**
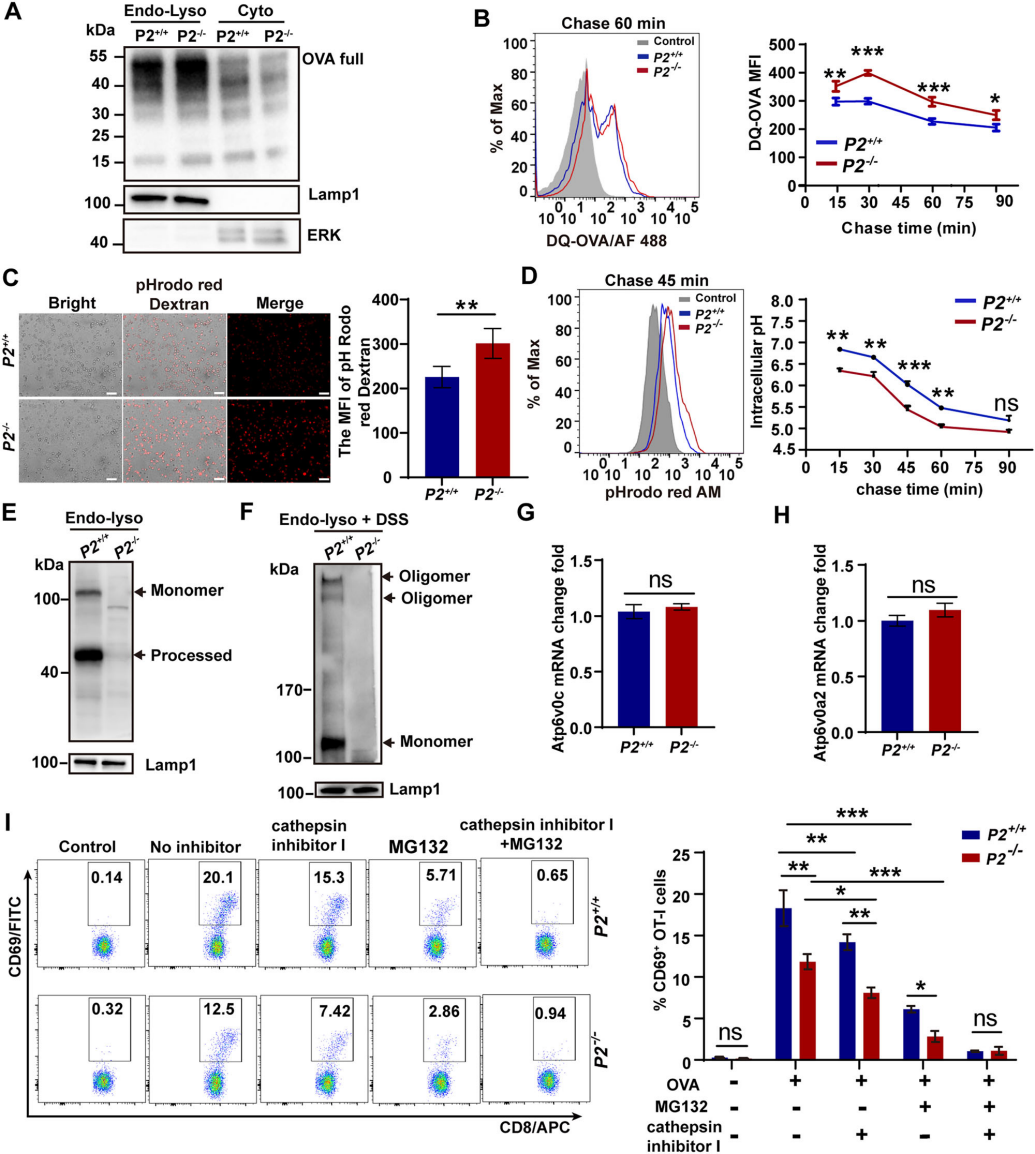
P2 inhibits excessive antigen degradation to enhance cross-presentation. **A** Immunoblot analysis of OVA degradation in separated endo-lysosomal and cytoplasmic fractions. **B** DQ-OVA proteolytic degradation in BMDCs measured by flow cytometry. **C**, **D** Endosomal acidification assessed by pHrodo red dextran and pHrodo red AM fluorescence using confocal microscopy (**C**) and flow cytometry (**D**). Scale bar, 50 μm. **E**, **F** Detection of P2 oligomerization in endo-lysosomal fractions without (**E**) or with DSS cross-linking (**F**). **G**, **H** mRNA expression of Atp6v0a2 and Atp6v0c in BMDCs. **I** CD8^+^CD69^+^ OT-I T cell activation after co-culture with BMDCs treated with OVA and proteasome inhibitor MG132 and/or cathepsin inhibitor. Data represent one of three independent experiments, and shown as mean ± SD. Statistically significant differences were estimated by two-way ANOVA with Šídák’s multiple comparisons test (**B**, **D**, **I**) and the unpaired two-tailed Student’s *t* test (**C**, **G**, **H**); ns not significant (*P* > 0.05); *P < 0.05, **P < 0.01, ***P < 0.001.

To further determine how P2 supports efficient cross-presentation following enhanced antigen uptake, we next examined its role in antigen processing and intracellular trafficking. Confocal microscopy of OVA-FITC-pulsed BMDCs revealed predominantly punctate fluorescence in *P2*^*-/-*^ cells, whereas *P2*^*+/+*^ BMDCs displayed a more diffuse cytoplasmic distribution of OVA-FITC (Fig. S8), consistent with enhanced redistribution of internalized antigen from endosomal compartments to the cytosol [3]. To investigate whether this difference in antigen distribution was associated with impaired access of internalized antigen to the cytosol, we performed immunoblot analysis of subcellular fractions. Compared with *P2*^*+/+*^ BMDCs, *P2*^*-/-*^ BMDCs accumulated increased amounts of OVA degradation fragments within endo-lysosomal compartments, whereas *P2*^*+/+*^ BMDCs showed greater accumulation of OVA-derived fragments in the cytosolic fraction (Fig. 6A). These results indicate that P2 deficiency is associated with reduced cytosolic availability of internalized antigen, consistent with previous reports [24]. We then asked whether the retention of antigen within endo-lysosomal compartments in *P2*^*-/-*^ cells would lead to its accelerated degradation. We employed DQ-OVA and observed that *P2*^*-/-*^ BMDCs exhibited significantly stronger fluorescence than *P2*^*+/+*^ BMDCs (Fig. 6B), indicating accelerated antigen degradation in the absence of P2.

Given that endosomal acidification tightly regulates antigen degradation [37], we hypothesized that P2 may modulate endosomal acidification to prevent premature antigen destruction. After a 30-min pulse with a pH-sensitive fluorescent probe (pHrodo red dextran), *P2*^*-/-*^ BMDCs displayed markedly stronger fluorescence signals (Fig. 6C), indicating enhanced endosomal acidification. To further quantify this, we measured the absolute endosomal pH in both genotypes. The results showed a gradual decrease in endosomal pH over time in both groups; however, *P2*^*+/+*^ BMDCs consistently maintained a higher pH than *P2*^*-/-*^ BMDCs (Fig. 6D). Notably, endosomal pH in *P2*^*-/-*^ BMDCs dropped into the range optimal for lysosomal protease activity (pH 4.5-5.0) [38]. To determine whether P2 localizes to these compartments and whether its absence is associated with altered endo-lysosomes pH, we isolated endo-lysosomal fractions and detected P2 by immunoblotting (Fig. 6E), consistent with our observation of P2 colocalization with EEA1 (Fig. 5H). Chemical crosslinking further revealed oligomeric P2 species within these fractions, demonstrating that P2 is present in endo-lysosomes in both monomeric and oligomeric forms (Fig. 6F). We then asked whether P2 influences acidification by altering the expression of the vacuolar ATPase (v-ATPase), the principal proton pump responsible for endosomal acidification [37]. No significant differences in the transcript levels of the core v-ATPase subunits Atp6v0a2 and Atp6v0c were observed between *P2*^*+/+*^ and *P2*^*-/-*^ BMDCs (Fig. 6G, H).

Finally, to functionally assess whether P2 supports a vacuolar versus cytosolic cross-presentation route, we investigated P2’s role in both routes using established inhibitors [39]. We assessed cross-presentation of BMDCs under conditions with MG132 (a cytosolic pathway inhibitor) and lysosomal cysteine proteases inhibitors (vacuolar pathway inhibitors). The combined application of MG132 and lysosomal cysteine proteases inhibitors completely inhibited cross-presentation, as indicated by the detection of CD69 on primed OT-I cells. Although cross-presentation was significantly impaired in the presence of MG132 or lysosomal cysteine proteases inhibitors alone in both groups of cells, significantly higher cross-presentation was still observed in *P2*^*+/+*^ BMDCs than in *P2*^*-/-*^ BMDCs, indicating that P2 is also involved in vacuolar pathway-mediated cross-presentation in addition to cytosolic pathway (Fig. 6I). We therefore infer that P2 prevents excessive antigen degradation, thereby enhancing cross-presentation.

## Discussion

Antigen cross-presentation is a critical process by which exogenous antigens are loaded onto MHC class I molecules to prime cytotoxic CD8^+^ T cells [2]. However, the efficiency of this process depends on multiple tightly regulated steps, including antigen uptake, endosomal processing, and pH homeostasis [37]. The present study elucidated that host PFP Perforin-2 (P2) enhances antigen cross-presentation by promoting antigen uptake and modulating antigen processing pathways. P2 facilitates the cross-presentation of both the model antigen OVA and tumor-associated antigens, thereby enhancing antigen-specific immune responses and promoting the clearance of tumor cells. These findings significantly enhance our understanding of the biological functions of PFPs, which are extensively distributed in vertebrates, particularly in relation to adaptive immunity.

Cross-presentation has been studied using various forms of antigens, such as soluble and particulate OVA [40, 41], which are internalized and processed through distinct pathways. However, the involvement of P2 in these processes has been debated. Ebrahimnezhaddarzi et al. reported that P2 mediates the cross-presentation of soluble rather than particulate OVA, whereas Rodríguez Silvestre et al. observed the opposite [23, 24]. In our study, P2 promoted the cross-presentation of both soluble and particulate OVA antigens. The most plausible explanation for these discrepancies lies in antigen dose, a key factor known to influence cross-presentation efficiency [41]. Consistently, we found that OT-I T cell proliferation was markedly higher in *P2*^*+/+*^ than in *P2*^*-/-*^ mice when exposed to low doses of either soluble or particulate OVA, but this difference disappeared at higher antigen concentrations (data not shown). Furthermore, adjuvants such as Poly I:C and LPS, which activate TLR3 and TLR4 respectively, can alter DC function and indirectly influence CTL priming [42, 43]. In their absence, the intrinsic role of P2 in antigen uptake becomes more apparent, emphasizing its importance in shaping CTL immunity. Although soluble and particulate antigens are processed through different routes, P2 enhances cross-presentation of both forms, suggesting a conserved function in optimizing antigen handling within DCs. Although our data support a critical role for P2 in antigen processing and cross-presentation, effective T cell activation requires not only antigen presentation and costimulation but also a third signal provided by DC-derived cytokines, which is essential for optimal T cell proliferation and effector differentiation [6]. Whereas P2 deficiency did not alter IL-6 secretion by BMDCs following LPS stimulation (Fig. S1J), the role of P2 in regulating the expression of cytokines such as IL-12 or IL-6 in DC-T cell coculture systems was not examined in this study. Therefore, it remains unclear whether P2-dependent regulation of cytokine expression impacts T cell proliferation or differentiation.

The hallmark of cellular immunity is the ability to produce an extremely diverse pool of CD8^+^ T cells [44]. Endogenous cytotoxic CD8^+^ T cells are powerful immune cells that kill infected cells or cancers [45]. OT-I CD8^+^ T cells express a transgenic TCR specific for the OVA_**257-264**_ peptide in the context of MHC class I [46], and this narrow specificity may not accurately represent the polyclonal nature of T cell responses in a physiological immune setting and the typical range of avidity within a T cell population [37, 47, 48]. To address this limitation, we exclusively employed DCs and CD8^+^ T cells derived from wild-type and P2 knockout mice, eliminating any influence from the TCR transgenic factor. Additionally, for in vivo experiments, antigen processing and presentation bypassed any potential artifacts from DCs isolation and culture confirming the contribution of P2-dependent cross-presentation to endogenous CD8^+^ T cell responses.

Surprisingly, upon immunization, we found that P2 deficiency not only affected endogenous CD8^+^ T cells but also led to a concomitant reduction in CD4^+^ T-cell responses. Because P2 is undetectable in T cells themselves, this effect likely arises secondarily from defective antigen presentation by DCs. Inadequate antigen preservation within over-acidified endosomes of *P2*^***-/-***^ DCs may impair MHC II loading efficiency and thus weaken CD4^+^ T-cell priming. Moreover, defective CD4^+^ T-cell activation can in turn dampen CD8^+^ T-cell expansion, as Th1-derived cytokines such as IL-2 and IFN-γ are critical for sustaining cytotoxic responses [49, 50]. Therefore, the impact of P2 on CD4^+^ T-cell responses may reflect its broader role in coordinating both MHC I and MHC II restricted antigen presentation.

Our tumor modeling data identify P2 as a critical regulator of antitumor immunity, whose absence impairs both the initiation and tumor-local execution of cytotoxic T-cell responses. The observed acceleration of tumor progression in P2-deficient hosts, together with reduced relative abundance of tumor-infiltrating CD8^+^ T cells and diminished ICOS expression, is consistent with defective activation and maintenance of effector T cells within the tumor microenvironment, rather than with enhanced Treg accumulation. The superior cross-presentation capacity observed in tumor-associated DCs from *P2*^*+/+*^ mice supports a DC-intrinsic contribution of P2 to antigen presentation. Nevertheless, the reproducible effects of P2 deficiency across multiple tumor models, argues for a broad and context-independent role of P2 in shaping tumor-associated antigen presentation and subsequent T-cell immunity. Effective antitumor immunity is strongly influenced by the efficient priming and expansion of tumor-specific CD8^+^ T cells within tumor-draining lymph nodes [1]. While the observed phenotypes are consistent with impaired cross-priming of tumor-specific CD8^+^ T cells, antigen-specific T cell priming in tumor-draining lymph nodes was not directly assessed in this study. Thus, defects in T cell expansion or tissue retention may also contribute to the observed phenotype, and future investigation of T cell cross-priming in tumor-draining lymph nodes may further clarify the role of P2 in antitumor immune regulation.

Despite the clear role of P2 in promoting APCs cross-presentation and CD8^+^ T-cell activation, several limitations should be acknowledged. This study utilized a global P2 knockout model rather than a DCs-specific deletion. Given that P2 is also expressed in macrophages and other phagocytes, we cannot completely exclude potential indirect effects arising from P2 deficiency in non-DC populations. Although our in vitro data demonstrated that P2 deletion did not impair macrophage-mediated antigen presentation, the possibility that macrophage-derived cytokines or altered tissue homeostasis may influence T-cell priming in vivo cannot be ruled out. Future studies employing CD11c-Cre - driven conditional knockout mice will be necessary to verify the cell-intrinsic role of P2 in DC-mediated antigen cross-presentation and tumor immunity.

Our study demonstrates that plasma membrane-associated P2 oligomers couple Ca^2+^ influx with ESCRT-mediated membrane repair and macropinocytic antigen uptake. ESCRT-dependent membrane repair is a conserved response to membrane disruption that is rapidly triggered by Ca^2+^ influx and mediated by the assembly of ESCRT-III polymers [36, 51]. This pathway has been well documented during mechanical injury, pathogen invasion, and pore-forming toxin attack [52], but its involvement in antigen uptake by immune cells has remained largely unexplored. We demonstrate that P2 oligomerizes on the plasma membrane in a pH-dependent manner, triggers Ca^2+^ signals, and initiates plasma membrane repair essential for macropinocytic antigen internalization. These findings extend the framework of “repair-associated endocytosis” to antigen acquisition [53], revealing that APCs exploit the plasma membrane repair machinery not only to restore membrane integrity but also to enhance antigen sampling in physiologically relevant contexts. Importantly, plasma membrane-localized P2 is primarily responsible for antigen uptake within immature APCs, which rely on macropinocytosis as their principal antigen uptake route [31]. As APCs mature, macropinocytosis is downregulated and receptor-mediated pathways predominate [31], consistent with the loss of P2 dependence we observed. Thus, P2 provides a selective advantage to immature APCs by integrating membrane repair with antigen internalization, maximizing sampling efficiency in peripheral tissues where antigen load and membrane stress are high. Although it is well established that an acidic extracellular pH promotes antigen internalization in APCs [54], the underlying mechanism has remained elusive. Our data establish that P2 mediates this pH-sensitive uptake. The potentiation of P2 oligomerization and Ca^2+^ influx under mildly acidic conditions suggests that inflammatory and tumor microenvironments may exploit this very mechanism to enhance P2 activity and, consequently, antigen internalization.

Although P2 is known to localize to multiple intracellular compartments [20], we observed that a subset of P2 exists in oligomeric forms within endosomes, suggesting that its structural state may contribute to the modulation of endosomal physiology. The markedly accelerated antigen degradation and lower endosomal pH in *P2*^*-/-*^ BMDCs indicate that P2 helps preserve a moderately acidic milieu that supports controlled antigen processing rather than full proteolysis. This function is mechanistically independent of transcriptional regulation of v-ATPase subunits, implying that P2 acts at the level of membrane or luminal regulation rather than proton pump expression. Notably, perforin-1 (the archetypal MACPF family protein) forms transmembrane oligomeric pores that enable granzyme B delivery into target cells [34, 35], illustrating that MACPF oligomers possess intrinsic structural capacity to alter membrane permeability and ion flux. By analogy, the presence of oligomeric P2 within endosomes may influence endosomal ionic or membrane properties in a manner that restrains over-acidification, although the precise mechanism remains to be determined.

**Fig. 7:**
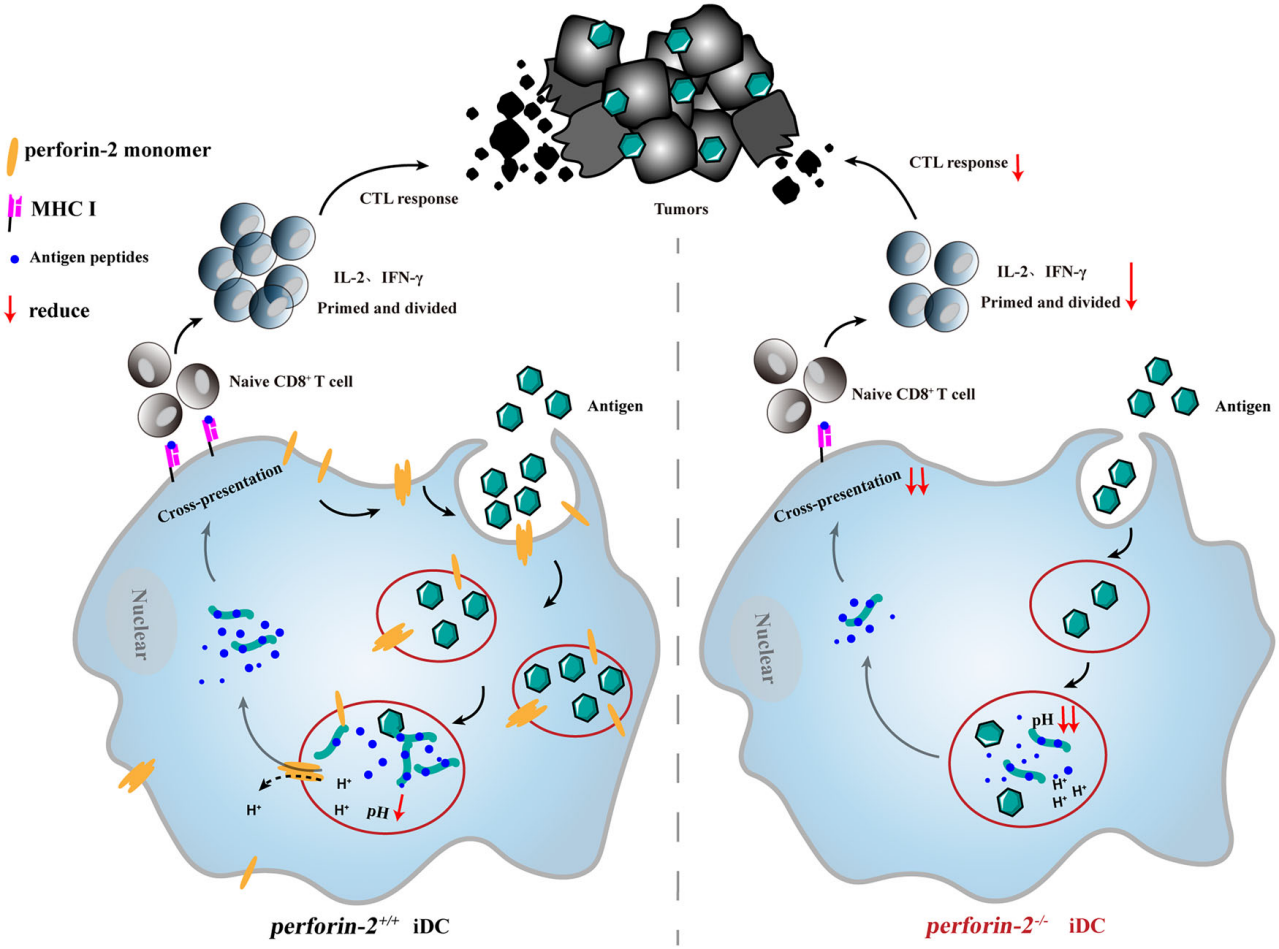
Model of P2-mediated membrane repair-driven antigen uptake and endosomal pH regulation that supports cross-presentation.

Our data suggest a model whereby P2 oligomers at the plasma membrane trigger Ca^2+^ influx to initiate membrane repair, thereby promoting macropinocytic antigen uptake, while within endosomes, P2 helps maintain a moderately acidic environment to prevent excessive antigen degradation and facilitate cross-presentation (Fig. 7). Collectively, our findings establish P2 as a crucial regulator of anti-tumor immunity by supporting efficient DCs cross-presentation

## Materials and Methods

### Mice

*P2*^***-/-***^ mice on a C57BL/6 background and OT-I CD8^**+**^ T cell receptor (TCR)-transgene mice were purchased from Cyagen Biosciences. In all experiments, age-matched male littermate mice (8–12 weeks old) were used for all experiments and randomly assigned to the indicated groups. For tumor challenge experiments, 6-8 mice were used per experiment, and each experiment was repeated twice. For other studies, sample sizes ranged from three to six mice, and these experiments were generally repeated twice. No statistical method was used to predetermine sample size; the sample sizes used in this study are consistent with those generally employed in the field and sufficient to detect reproducible biological differences. All mice were maintained under specific pathogen-free conditions. All animal studies were performed in accordance with procedures approved by the Animal Ethics Committee of the Kunming Medical University.

### Cell lines

B16/F10 melanoma cells of C57BL/6 origin (FuHeng Biology) were cultured in Dulbecco’s Modified Eagle’s medium (DMEM; BasalMedia) supplemented with 10% heat-inactivated fetal bovine serum (FBS; ExCell Bio). B16/F10-OVA cells, an ovalbumin (OVA)-expressing B16/F10 cell line (FuHeng Biology), were cultured in Roswell Park Memorial Institute 1640 medium (RPMI 1640; Gibco) with 10% heat-inactivated FBS. All cell lines were identified by STR typing, and all mycoplasma contamination tests were negative.

### Flow-cytometry analysis of surface markers

Surface staining was performed using the following antibodies: CD8a-APC (100711) or PE (100707) or FITC (100705), CD4-FITC (100405) or APC (100411), CD3-APC (100235) or FITC (100203), CD45-Pacific Blue (103126), CD69-FITC (104505), TCR V2α-PE (12581280), CD11c-Pacific Blue (117322), CD86-FITC (105005), CD40-FITC (102905), CD80-APC (104713), 25-D1.16-PE (12-5743-82), H-2K^b^ (MHC I)-APC (116517), I-A/I-E (MHC II)-FITC (MA1-10403), NK1.1-FITC (108705), CD19-FITC (115505), F4/80-PE (111603), PD1-APC (135209), ICOS-Percp-Cy5.5 (117423), Foxp3-PE (126403), CD25-APC (113708), XCR1-Alexa Fluor 700 (148243), SIRPα-Percp-Cy5.5 (144009) and CD24-PE (138503). All antibodies were diluted according to the manufacturer’s instructions. Except for the I-A/I-E (MHC II)-FITC, TCR V2α-PE and 25-D1.16-PE antibodies, which were purchased from Thermo Fisher, all other antibodies were purchased from Biolegend. Stained cells were detected using a FACS Celesta flow cytometry (BD Biosciences) and analyzed with FlowJo 10.8.1 software.

### Generation of BMDCs and maturation assays

BMDCs were generated as previously described [25]. Briefly, femur and tibia from mice were isolated and bone marrow cells were collected. Single-cell suspensions were cultured at a density of 4 × 10^6^ cells in Petri dishes containing 10 ml of complete RPMI 1640 supplemented with 20 ng/ml rmGM-CSF (PeproTech, 315-03-20), 1% non-essential amino acid (Gibco, 11140050), 1% sodium pyruvate (Gibco, 11360070), 0.1% β-mercaptoethanol (Gibco, 21985023), 0.1 mg/ml streptomycin and 100 U/ml penicillin (BasalMedia, S110JV). On day 3, an additional 10 ml of complete RPMI 1640 containing 20 ng/ml rmGM-CSF was added. Non-adherent cells were collected on day 6 of culture for subsequent experiments. Day 6 BMDCs were used for all in vitro assays unless stated otherwise. For maturation test, BMDCs were inoculated in 24-well plates (2.5 × 10^5^/well) and pulsed with 1 µg/ml LPS (Sigma-Aldrich, SMB00610-1MG) for 18 h. The surface costimulatory molecules on BMDCs were analyzed by flow cytometry, and the concentration of IL-6 (Novus Biologicals, M6000B) in the culture supernatants was measured by ELISA.

### Generation of bone marrow-derived macrophages (BMDMs)

BMDMs were generated as previously described [55]. Briefly, bone marrow (BM) cells were collected from sex- and age-matched *P2*^*+/+*^ and *P2*^*-/-*^ littermates (8–12 weeks old). 10% FBS DMEM medium containing 20% L929 cell supernatant, 0.1 mg/ml streptomycin, and 100 U/ml penicillin was added. On days 3 and 5, 5 mL of fresh DMEM complete medium containing 20% L929 cell supernatant was added, respectively. Cells were cultured until day 7 for P2 expression detection and cross-presentation experiments.

### MHC I-restricted cross-presentation assay

The BMDCs were incubated with 2 mg/ml soluble OVA (Sigma, A5503-1G) or 5 μg/ml OVA_**257-264**_ peptide (Anlianpeptide Co.,Ltd) for 3 or 18 h. The cells were then washed and stained with PE-conjugated 25-D1.16 mAb, which specifically recognizes H-2K^b^-OVA_**257-264**_ complexes. These complexes were quantified by flow cytometry.

### Preparation of particulate OVA antigen

OCSs were generated with slight modifications from previously described method [56]. Briefly, splenocytes from C57BL/6 wild-type mice were collected, processed into a single-cell suspension, and treated to lyse red blood cells. The mouse CD3ε MicroBead Kit (Miltenyi Biotec, 130094973) was used to remove total T cells from the spleen (Fig. S9). OVA (10 mg/ml) was added to the cells and incubated 37 °C for 10 min. The cells were washed with pre-cooled PBS to remove excess OVA proteins and then irradiated with 200 mJ/cm^2^ UVC. The OVA-coated splenocytes (OCSs) were used as particulate OVA antigen in subsequent experiments.

### OT-I T cell labeling

Staining was performed with a CellTrace Violet (CTV) Proliferation Kit (Thermo Fisher, C34571) according to manufacturer’s instructions. Briefly, the OT-I T cells were isolated using the mouse pan T cell isolation kit II (Miltenyi Biotec, negative sorting, 130-104-075) and resuspended in 2.5 μM CTV solution at 1 × 10^6^ cells/ml, followed by incubation at 37 °C for 5 min in the dark. The labeling was terminated by adding media with 10% FBS and incubating for another 5 min. Cells were centrifuged at 300 g for 5 min, resuspended in RPMI 1640 media, and incubated at 37 °C for 10 min.

### Antigen cross-presentation assays in vitro

For the cross-priming assay [57], 5 × 10^4^ BMDCs or primary splenic cDC1 and cDC2 subsets were seeded in a U-bottom 96-well plate and incubated with varying amounts of OVA for 3 h, or with different doses of OCSs for 20 h. Subsequently, 1 × 10^5^ OT-I T cells were co-cultured for 16 h, and T cell surface activation marker CD69 was detected by flow cytometry. Additionally, concentrations of IL-2 (Novus Biologicals, VAL602) and IFN-γ (Novus Biologicals, VAL607) in the supernatant were measured by ELISA. In parallel, BMDCs incubated with OVA alone (without OT-I T cells) were included as controls to assess cytokine release from BMDCs under antigen stimulation. For the BMDMs cross-activation assay, BMDMs were stimulated with 0.1 mg/ml OVA for 3 h, followed by washing twice with PBS. OT-I was then added and co-cultured for 18 h, and CD69 molecules were detected by flow cytometry.

For T cell proliferation assay, CTV-labeled OT-I T cells were added to the antigen-stimulated BMDCs and cultured at 37 °C and 5% CO_2_ for 72 h to detect CTV dilution by flow cytometry. In a separate experiment, BMDCs were incubated with 0.1 mg/ml OVA for 3 h, in the presence of either 5 μM proteasome inhibitor MG132 (Absin, ABS817874), 20 μM Cathepsin Inhibitor I (Selleck, s2847-5mg), or both, or neither [39]. The cells were washed and co-cultured with OT-I cells for 16 h, and flow cytometry was used to detect the CD8^**+**^CD69^**+**^ T cells.

### Cytotoxicity assay

Soluble OVA and OCS were used as the immunogen. In vitro cytotoxicity assays were performed as described [28].

### OT-I cells proliferation in vivo

In vivo OT-I proliferation assays were conducted with slightly adjustment from previously described [23, 24]. Briefly, 1 × 10^6^ CTV-labeled OT-I T cells were injected intravenously (i.v.) into mice. Twenty-four hours later, mice received 20 µg soluble OVA or 2 × 10^6^ OCS via i.v. injection. After sixty-four hours of antigen immunization, spleens were harvested, and the single-cell suspensions were analyzed by flow cytometry.

### Intracellular cytokine staining

Briefly, cells were stimulated with 10 μg/ml OVA_**257-264**_ or OVA_**323-339**_ or gp100_**25-33**_ (gp100 protein residues 25–33) (both Anlianpeptide Co.,Ltd) for 12 h. Next, the cells were washed once with PBS and fixed with 4% paraformaldehyde for 20 min. After penetration with 0.1% triton X-100, the cells were incubated with 2 µg/ml IFN-γ-APC (Biolegend, 505809) at room temperature for 40 min.

### Endogenous T cell responses

For the soluble OVA immunogen, mice were immunized with 0.5 mg OVA once a week for 5 weeks. For the OCS immunogen, 1 × 10^7^ OCS were injected i.v. into mice for one week. After the last antigens stimulation, CD8^+^ T and CD4^+^ T cells in the spleens were detected by flow cytometry, and IFN-γ within these cells was detected as described above. Specific proliferation of endogenous CD8^+^ T cell was assessed in ex vivo as previously described [46].

### Antigen-specific CTL assay

CTL assays were only slightly adjusted as previously mentioned in vivo [58]. Briefly, total splenocytes of naive C57BL/6 mice were pulsed with or without 1.5 µg/ml OVA_**257-264**_ for 1 h at 37 ˚C. Subsequently, pulsed cells were labeled with 10 µM CTV (CTV^high^) for 10 min at 37 ˚C and washed twice. Unpulsed cells were simultaneously labeled with 1 µM CTV (CTV^low^). Cells were mixed at a 1:1 ratio and total 2 × 10^7^ cells were injected intravenously into mice that had been challenged with soluble OVA or OCS. The mice were sacrificed after 28 h, and the total events corresponding to both fluorescent intensities (CTV^low^ and CTV^high^) in spleen were determined by flow cytometry. The percentage lysis of OVA_**257-264**_ pulsed splenocytes was calculated as follows: percentage lysis = 100-(CTV^high^/ CTV^low^) _immunized_ × 100 × (CTV^low^/ CTV^high^) _unimmunized_.

### Tumor models

For B16/F10-OVA tumor model, mice received 0.5 mg OVA subcutaneously immunization on day 0 and 7. On day 14, 3 × 10^5^ B16/F10-OVA melanoma cells were subcutaneously incubated on the right flank. For B16/F10 tumor model, based on the previous description and make mildly modifications [30]. Briefly, 5 × 10^6^ B16/F10 cells were repeatedly freeze-thawed and inoculated into the subcutaneously. 14 days after immunization, mice were challenged with 3 × 10^5^ live B16/F10 cells on the right flank. Tumor growth was monitored every 2–3 days by measuring the two largest perpendicular diameters with a caliper, and tumor volume was calculated using the formula: *length × width*^*2*^*/2* and expressed as mm^3^ [59]. The mouse experiment was terminated when the tumor grew to day 20 or the tumor diameter reached 20 mm, or the tumor size reached 2000 mm^3^. For flow cytometric analysis of tumor-infiltrating cells, tumors were harvested after 20 days of growth. Approximately 200 mg of tumor tissue from each mouse was minced and dissociated into a single-cell suspension, passed through a 70 μm cell strainer, washed with PBS buffer containing 2 mM EDTA, and subjected to antibody staining. In parallel, splenocytes from tumor-bearing mice were isolated for comparative analysis. The frequencies of ICOS^+^ and PD-1^+^ T cells, as well as regulatory T cells (Tregs; CD4^+^CD25^+^Foxp3^+^), were analyzed in both spleen and tumor samples by flow cytometry.

### Antigen uptake assays

For in vitro antigen uptake, day 6 BMDCs were pre-treated with 5 μM ML240 (Proteintech, 1346527-98-7) or 50 μM 5-(N-ethyl-N-isopropyl) amiloride (EIPA; MedChemExpress, HY-101840A) or 20 μM wortmannin (CSNpharm, 19545-26-7) or left untreated for 30 min at 37 °C, followed by the addition of 20 μg/ml FITC-OVA (BOSTER, bs-0283P) or 100 μg/ml dextran (Sigma-Arich, 46945-100MG-F) for 15 min. Cells incubated with FITC-OVA or FITC-dextran at 0 °C served as controls. In a separate experiment, BMDCs were resuspended in normal (pH 7.4) or acidified (pH 6.5) medium, and treated with 20 μg/ml FITC-OVA at 37 °C for 15 min. The percentage of cells taking up OVA or dextran was detected by flow cytometry. The intracellular distribution and fluorescence intensity of OVA and dextran were observed using an Olympus FV4000 confocal laser microscope system and quantified using ImageJ software.

For in vivo antigen uptake, it was as described previously [60].

### Cytomembrane P2 detection

The distribution of P2 on the cytomembrane of BMDCs was detected by flow cytometry as previously described [22]. A polyclonal rabbit anti-mouse/rat P2 IgG antibody (Thermo Fisher) was used. The secondary antibody was FITC donkey anti-rabbit IgG (Biolegend, 406403) at 1/1000 dilution.

### Immunofluorescence and confocal microscopy

For immunofluorescence staining, BMDCs were seeded onto 15 mm glass-bottom confocal dishes and cultured overnight. Cells were incubated with Hanks’ Balanced Salt Solution (HEPES, Gibco, 15630080) adjusted to pH 7.4 or pH 6.5 for 15 min at 37 °C to mimic physiological and mildly acidic conditions. Cells were then fixed with 4% paraformaldehyde for 15 min at room temperature, permeabilized with 0.3% Triton X-100 for 10 min (cells were not permeabilized when observing the cell surface P2), and blocked with 5% normal goat serum for 1 h. Primary antibodies were added and incubated overnight at 4 °C, followed by Alexa Fluor 647-conjugated secondary antibodies (proteintech, 1:1000 dilution) for 1 h at room temperature in the dark. Nuclei were counterstained with DAPI (Thermo Fisher, 62248). Images were captured using an Olympus FV4000 confocal laser scanning microscope.

For colocalization analyses, BMDCs were first pulsed with 20 μg/ml FITC-conjugated OVA for 15 min at 37 °C under pH 7.4 or pH 6.5 conditions, followed by washing with ice-cold PBS to remove extracellular antigen. Cells were then stained with antibodies against P2 (Cloud-Clone, PAB352Mu01), EEA1 (Cell Signaling Technology, 3288 T), and OVA. Fluorescence intensity and colocalization coefficients were quantified using ImageJ.

### Calcium flux analysis by live-cell fluorescence imaging

To monitor intracellular calcium dynamics, BMDCs were loaded with Fluo-4 AM (Invitrogen, 1 μM, F14217) in HEPES containing 10 mM Probenecid (Proteintech, 57-66-9) for 30 min at 37 °C in the dark, followed by a 30-min de-esterification period in dye-free medium. Cells were then transferred to glass-bottom dishes and imaged in HEPES-buffered solution (pH 7.4 or pH 6.5) that either contained or lacked Ca^2+^. Time-lapse fluorescence imaging was performed using an Olympus FV4000 confocal microscope (excitation: 488 nm; emission: 510–540 nm). Baseline fluorescence was recorded for 30 s, after which cells were stimulated with HEPES solution containing OVA (20 μg/ml) under the indicated conditions (pH 7.4 or 6.5). Images were acquired every 3 s for up to 200 s. Changes in intracellular fluorescence intensity were analyzed using ImageJ.

### Antigen proteolysis, and endosomal pH analyses

To assess antigen proteolysis, 3 × 10^5^ BMDCs were pulsed with 10 μg/ml DQ-OVA (Thermo Fisher, D12053) at 37 °C for 10 min. The cells were then washed twice, resuspended in medium without FBS, and chased for 15, 30, 60 and 90 min. At each time point, cells were kept on ice, and fluorescence was measured by flow cytometry. As a control, BMDCs were pulsed with DQ-OVA for 10 min at 4 °C, washed twice, and analyzed by flow cytometry.

To assess endosomal pH, BMDCs were cultured in 15 mm glass-bottom dishes, and treated with 50 μg/ml pHrodo^TM^ red dextran (Life technologies, P10361). The cells were incubated at 37 °C for 30 min, and washed three times with PBS. Subsequently, cells were analyzed by Olympus FV4000 confocal laser microscope system. For quantitative measurement of endosomal pH, BMDCs were loaded with 50 μg/ml pHrodo AM (Thermo Fisher, P35372) under the same conditions and analyzed by flow cytometry according to the manufacturer’s instructions. Briefly, after incubation, cells were washed, resuspended in PBS, and analyzed using a flow cytometer at 15, 30, 45, 60 and 90 min. To calibrate fluorescence to absolute pH values, a pH calibration curve was generated using pH calibration buffers ranging from pH 4.5 to 7.5 containing 10 μM nigericin and 10 μM monensin to equilibrate intra- and extracellular pH. Fluorescence intensity at each calibration point was recorded, and the corresponding pH of experimental samples was determined by interpolation from this standard curve using FlowJo.

### Western blot analysis

#### Assessment of P2 expression

*P2*^*+/+*^ and *P2*^*-/-*^ mouse-derived BMDCs, cDC1, cDC2 and BMDMs were used to validate P2 expression (Abcam, Ab25146). To detect P2 expression in T cells, spleens were collected from OCS-immunized or non-immunized mice, and total CD3^+^ T cells were sorted using positive and negative magnetic beads for P2 expression detection.

#### P2 oligomerization assays

For oligomerization studies, BMDCs were stimulated at pH 7.4 or 6.5 for 15 min. Plasma membrane (Invent, SM-005) or endosome (Invent, ED-028) fractions were isolated using a commercial subcellular fractionation kit according to the manufacturer’s protocol. The membrane fraction was then treated with the chemical cross-linker disuccinimidyl suberate (DSS; Thermo Fisher, 21655) prior to electrophoresis to stabilize protein complexes.

#### Antigen degradation assays

The antigen degradation assay was performed as previously described with minor modifications [9]. Briefly, BMDCs were pulsed with 200 μg/ml OVA at 4 °C for 15 min. After washing, internalization was initiated by chasing the cells at 37 °C for 1 h. Subsequently, endosomal and cytoplasmic fractions were isolated for immunoblot analysis. Full-length OVA ( ~ 46 kDa) and its degradation products were detected by immunoblotting.

#### General immunoblot procedure

Cell lysates or subcellular fractions were resolved by SDS-PAGE and transferred to membranes. The following antibodies were used for detection: polyclonal rabbit anti-mouse/rat P2, monoclonal goat anti-β-actin (66009-1-Ig), anti-Lamp1 (late endosome marker; Abcam, AB125068), and anti-ERK (cytoplasmic marker; Abcam, AB201015). Horseradish peroxidase (HRP)-conjugated goat anti-rabbit/mouse IgG (H + L) (Proteintech) was used as the secondary antibody.

### RNA isolation and quantitative real-time PCR (qRT-PCR)

qPCR was performed using TB Green Premix Ex Taq II (Takara Bio) on a QuantStudio 5 system strictly according to the manufacturer’s instructions, and relative Atp6v0a2 and Atp6v0c mRNA levels were normalized to GAPDH using the 2^–ΔΔCt^ method. The sequences of the primer pairs used were as follows: Atp6v0a2: Forward: 5’-CCTATGCAGAGCTGGACGAG-3’, Reverse: 5’-CCGACTCCATAGGCATCCAC-3’; Atp6v0c: Forward: 5’-GATCTTCGCTTGCCTCCTCG-3’, Reverse: 5’-CCACTCTTGGCTGTGCCAT

A-3’; GAPDH: Forward: 5’-AACTTTGGCATTGTGGAAGG3’, Reverse: 5’-GGATGCAG GGATGATGTTCT-3’.

### Statistical analysis

All statistical analyses were perform*ed* with GraphPad Prism 9.4.1 software using the unpaired two-tailed Student’s *t* test, the log-rank test and two-way ANOVA with Šídák’s multiple comparisons test. *P* values greater than 0.05 are considered non-significant and expressed as *P* > 0.05, while those less than 0.05 are considered significant and are denoted as *P < 0.05, **P < 0.01, ***P < 0.001.

## Supplementary information

Original Data

Supplementary Material

## Data Availability

All data needed to evaluate the conclusions in the paper are present in the paper and/or the Supplementary Materials.
