# Supplementary material for: Perforin-2 enhances antigen-specific CTL immune response by promoting cross presentation
Source: Cell Death Dis. 2026 Apr 9;17(1):485. doi: 10.1038/s41419-026-08705-1 (PMC13187254; doi:10.1038/s41419-026-08705-1)

Original western blot images for Fig. S1D

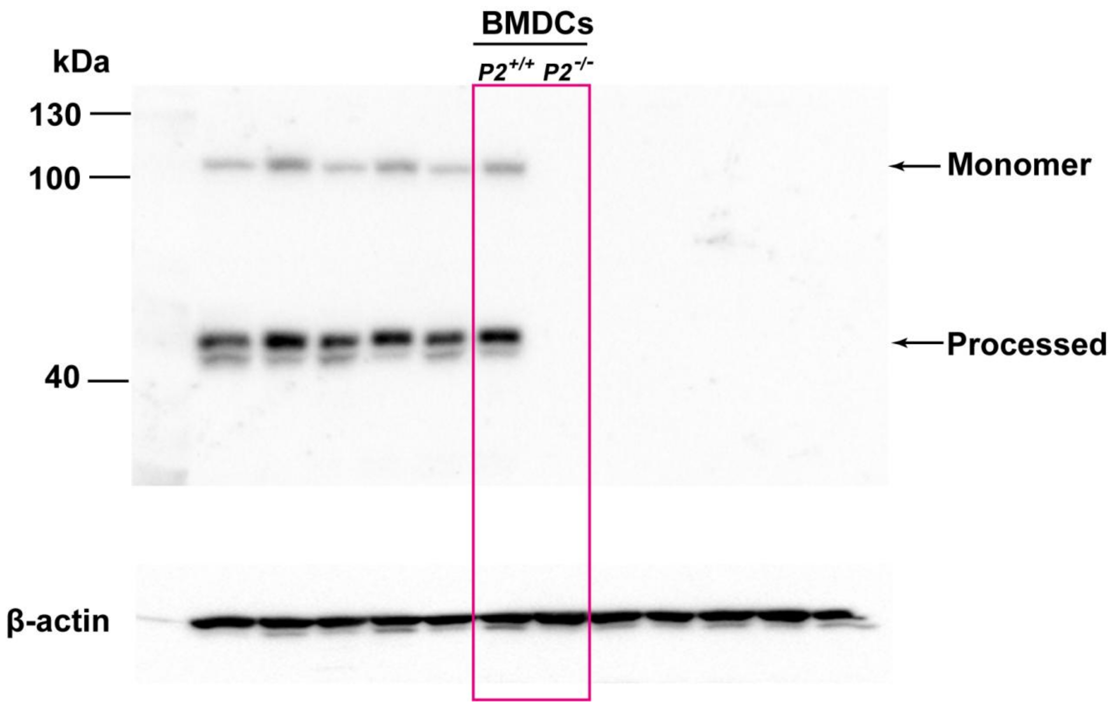

Original western blot images for Fig. S2H

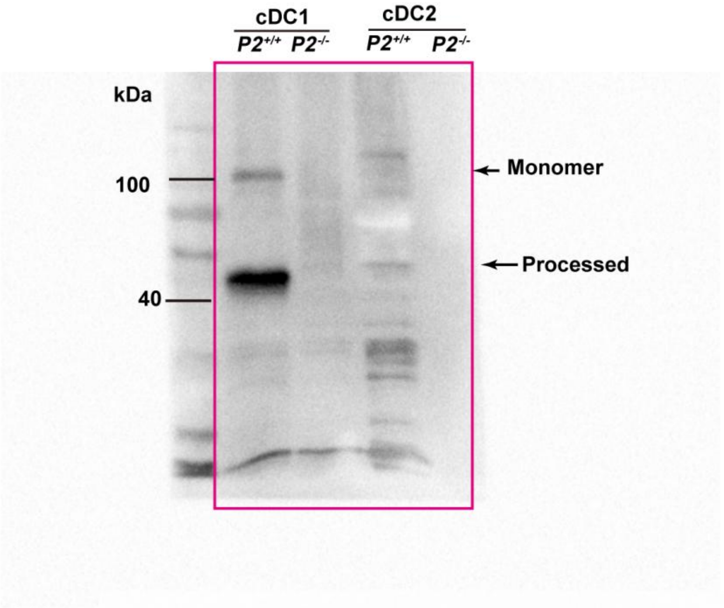

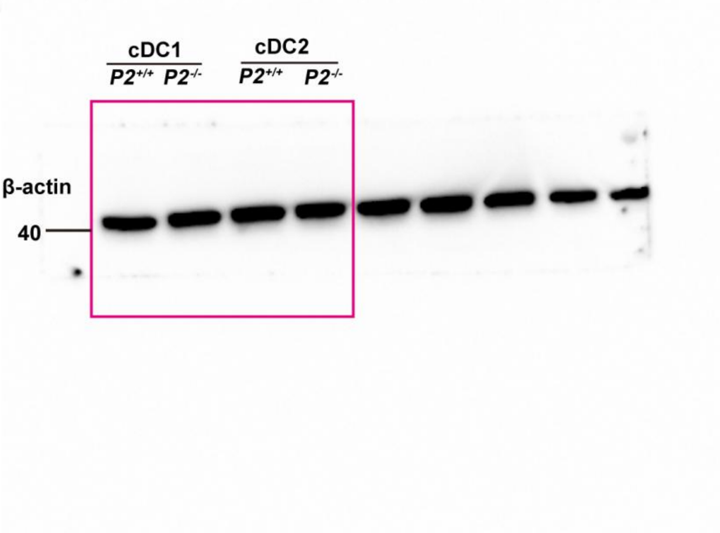

Original western blot images for Fig. S2K

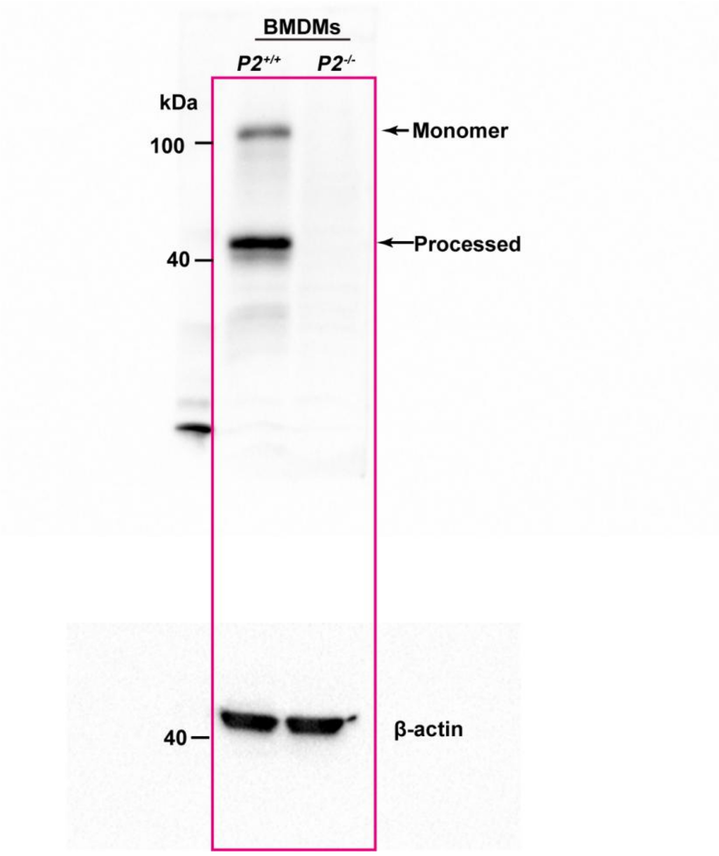

Original western blot images for Fig. S4G

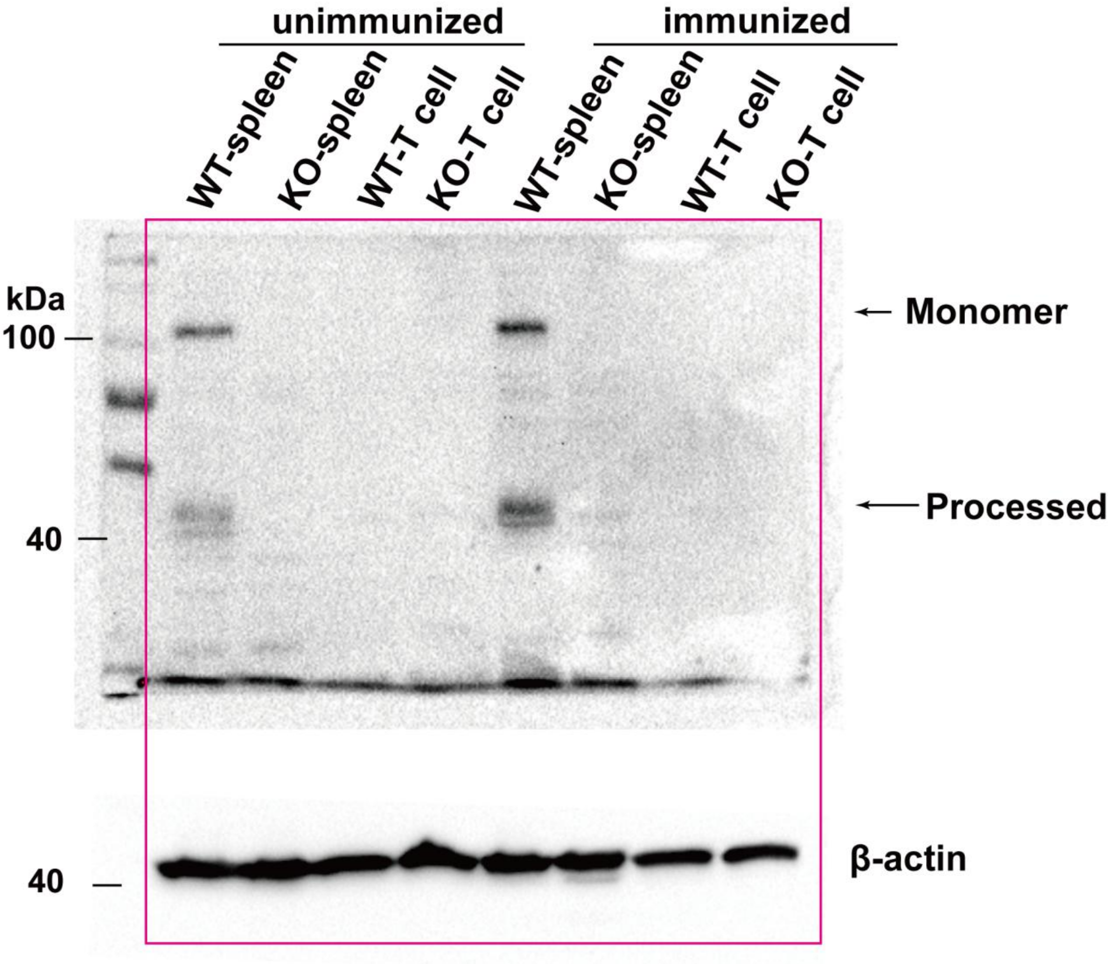

Original western blot images for Fig. 5I

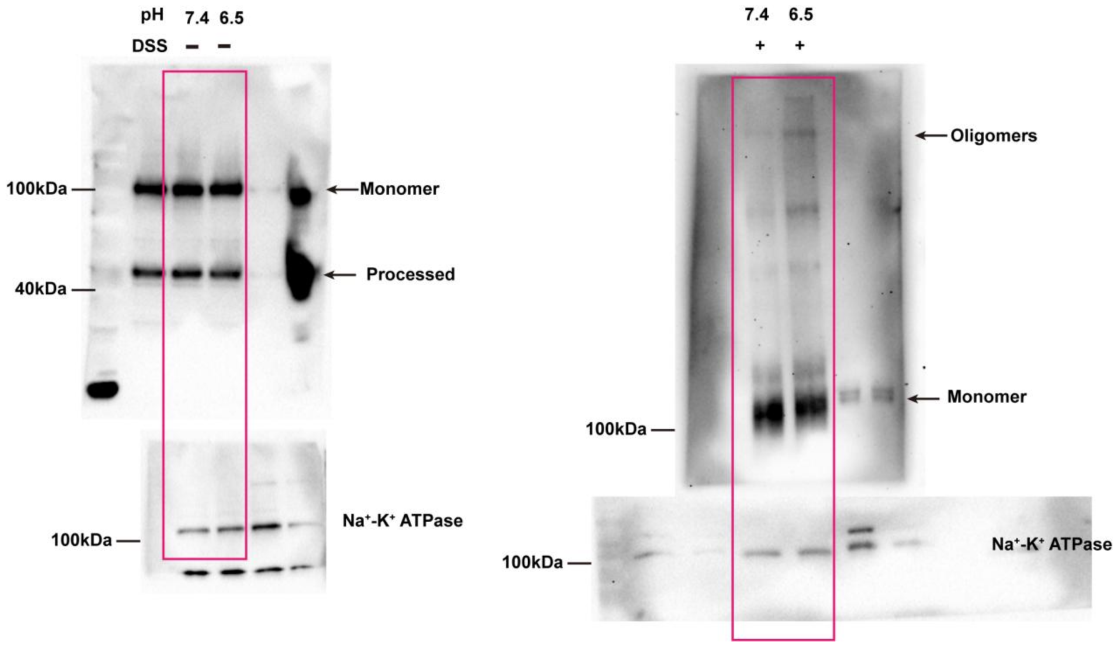

Original western blot images for Fig. 6A

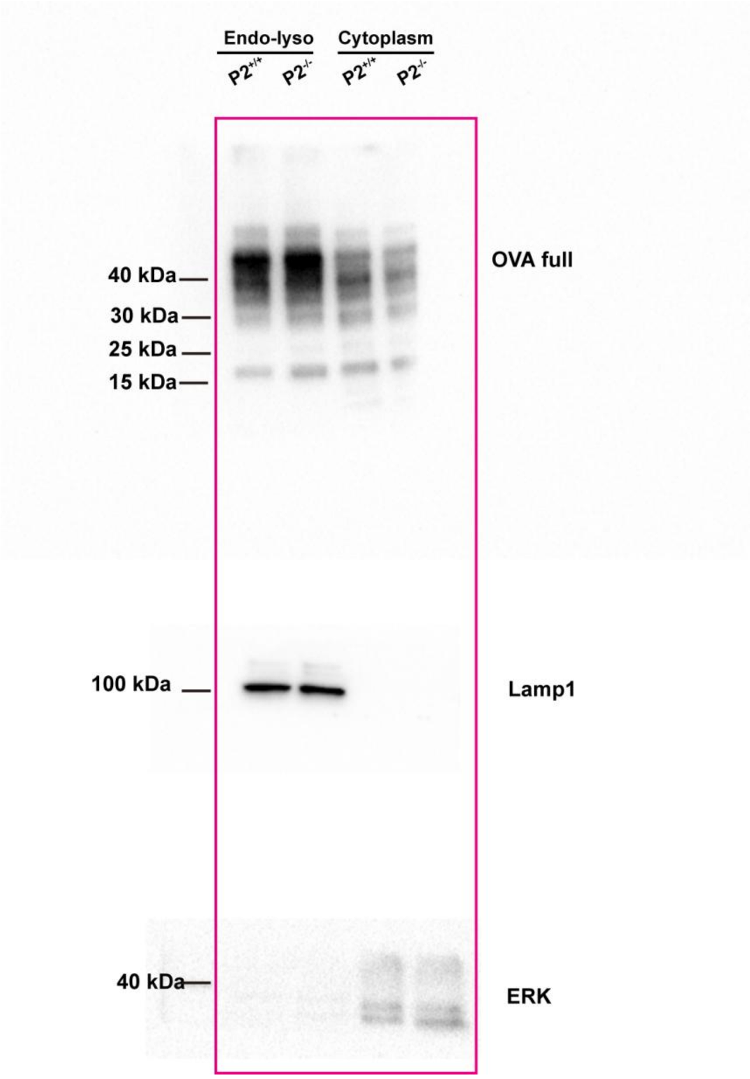

Original western blot images for Fig. 6E

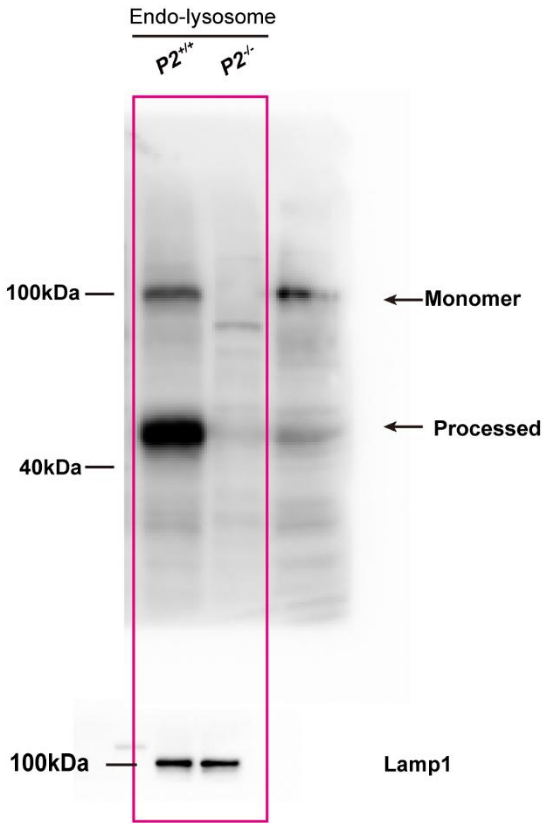

Original western blot images for Fig. 6F

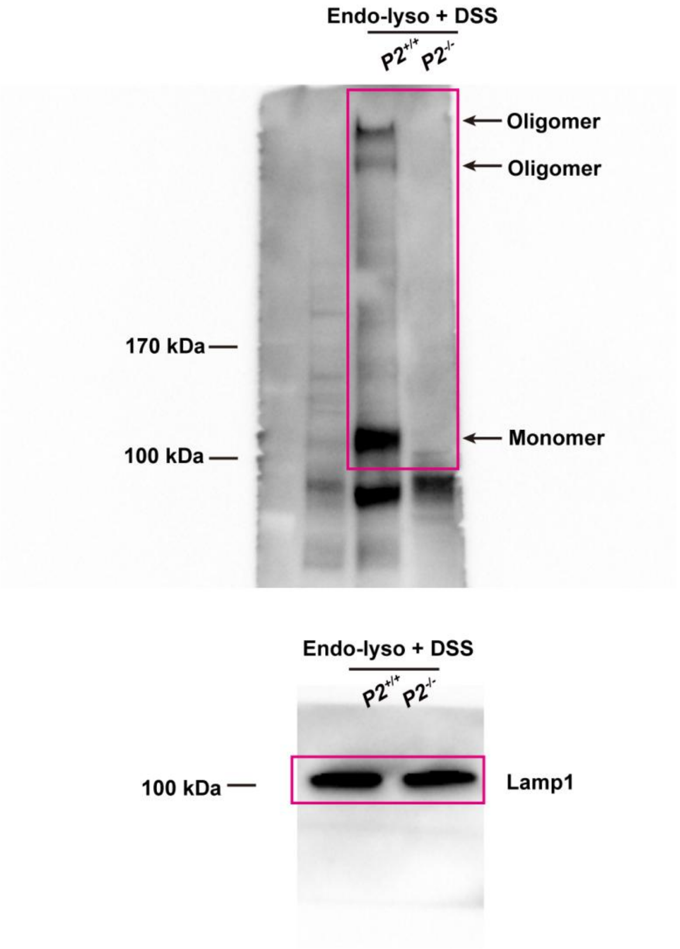

Supplement: Supplementary file 1 — Original Data [file 41419_2026_8705_MOESM1_ESM.pdf]
