# Supplementary material for: Perforin-2 enhances antigen-specific CTL immune response by promoting cross presentation
Source: Cell Death Dis. 2026 Apr 9;17(1):485. doi: 10.1038/s41419-026-08705-1 (PMC13187254; doi:10.1038/s41419-026-08705-1)
Supplement: Supplementary file 2 — Supplementary Material [file 41419_2026_8705_MOESM2_ESM.docx]

**Supporting Information**

Perforin-2 enhances antigen-specific CTL immune response by promoting cross presentation

Zhi-kai Zha^1, #^, Cheng-jie Deng^2, #^, Ling-jun Shen^3, #^, Ling-zhen Liu^1, #^, Yun-xiao Huang^1^, Yan-hong Li^1^, Li Lv^1^, Ke Zhang^1^, Lin-shuang Chen^1^, Fei-er Chen^1^, Sheng-an Li^1, 4,^ *

^1^ Yunnan Provincial Key Laboratory of Public Health and Biosafety & Department of Pathogen Biology and Immunology, Faculty of Basic Medical Science, Kunming Medical University, Kunming, Yunnan 650500, China

^2^ The Center of Experimental Teaching, Faculty of Basic Medical Science, Kunming Medical University, Kunming, Yunnan 650500, China

^3^ Department of Tuberculosis, Yunnan Clinical Medical Center for Infectious Diseases, the Third People's Hospital of Kunming (The Sixth Affiliated Hospital of Dali University), Kunming 650041, China

^4^ Key Laboratory of Sepsis and Brain Regulation in Universities of Yunnan Province, Faculty of Basic Medical Science, Kunming Medical University, Kunming, Yunnan 650500, China

^#^ These authors contributed equally to this work

* Corresponding author

Email: [lishengan@163.com](mailto:lishengan@163.com) (Sheng-an Li)

**This file includes:**

Figures S1 to S9 and original Western blot data

**Data and figures**


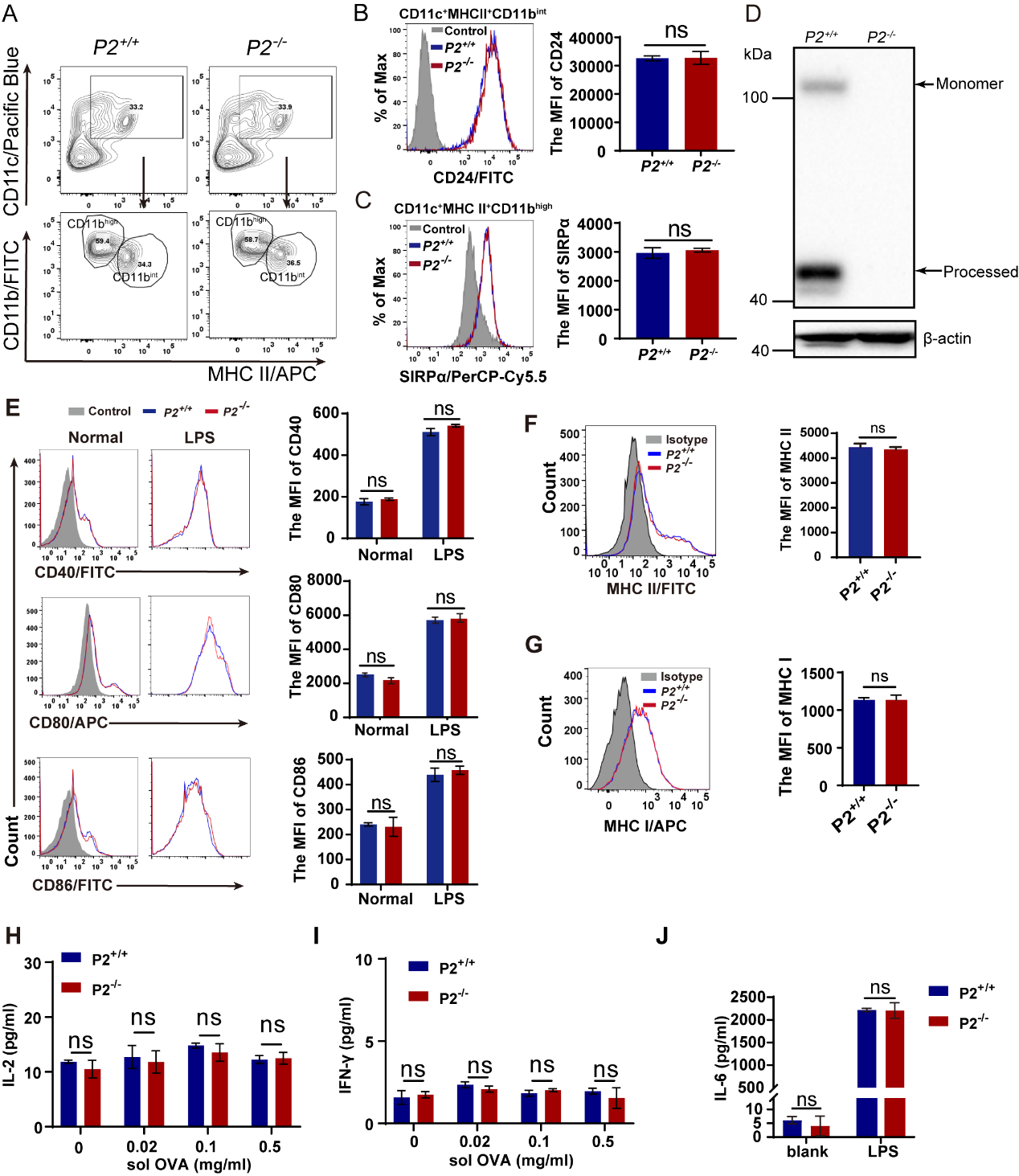


**Figure. S1.** P2 deficiency does not affect the differentiation and maturation of BMDCs. **(A)** Gating strategy for CD11c^+^MHC II^+^CD11b^int^ and CD11c^+^MHC II^+^CD11b^high^ BMDCs. CD11b^int^ and CD11b^high^ represent intermediate and high expression levels of CD11b, respectively. **(B and C)** Expression of CD24 and SIRPα in gated BMDCs. **(D)** Western blot detection of P2 expression. **(E)** Surface expression of costimulatory molecules (CD40, CD80, CD86) on LPS-stimulated BMDCs. **(F and G)** MFI of MHC class II and class I molecules on unstimulated BMDCs. **(H and I)** IL-2 and IFN-γ concentration in supernatant of BMDCs by treated with increasing OVA concentrations. **(J)** IL-6 concentration in supernatant of BMDCs after LPS stimulation. Data are presented as mean ± SD (n = 3); statistical analysis was performed using an unpaired two-tailed Student’s *t* test (B, C, F and G) and two-way ANOVA with Šídák's multiple comparisons test (E, H-J); ns, not significant (*P* > 0.05). All data are representative of two independent experiments.


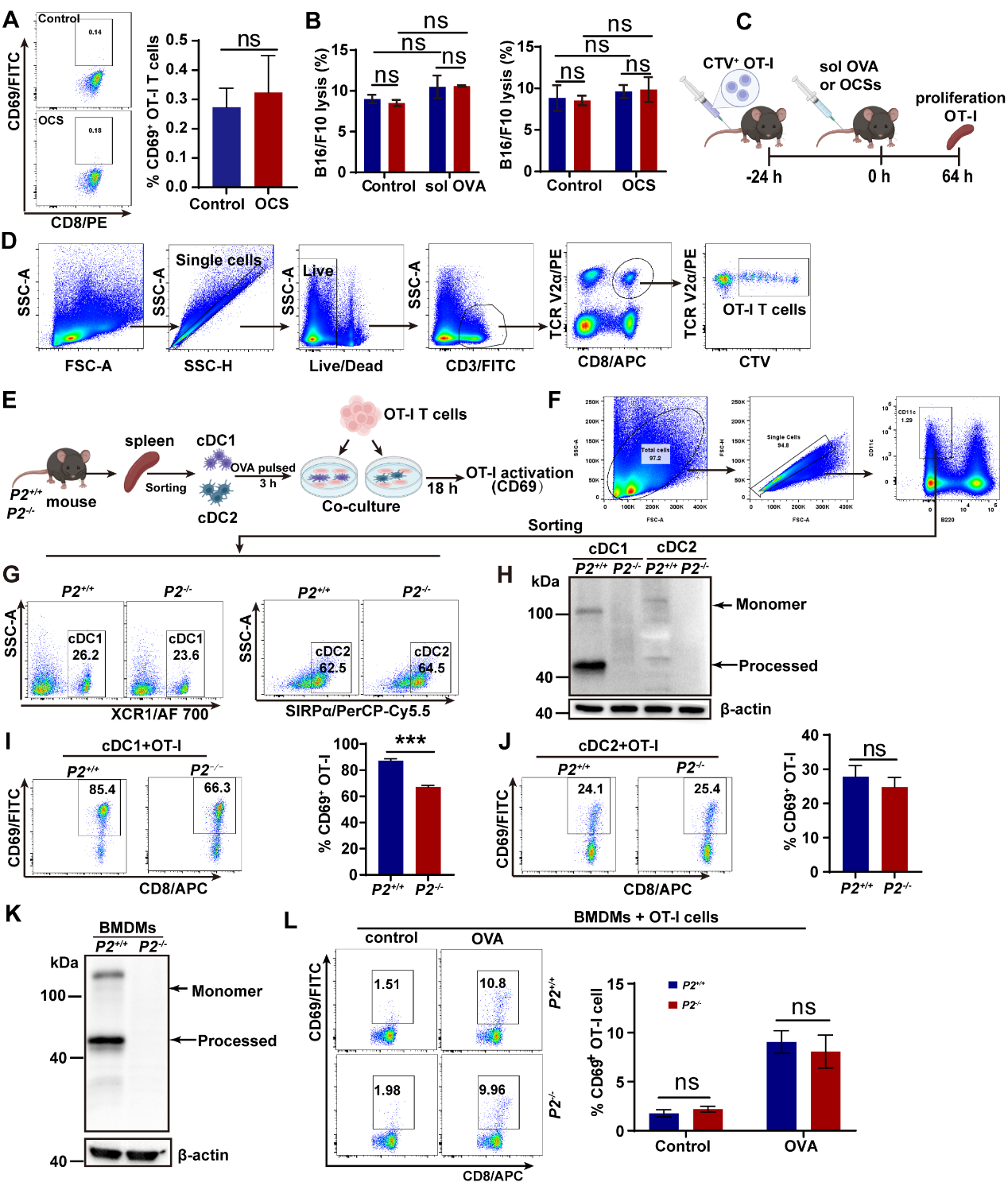


**Figure. S2.** P2 regulates cross-presentation by dendritic cells to CD8^+^ T cells. **(A)** CD69 expression on OT-I T cells co-cultured with UV-irradiated OCSs. **(B)** Specific lysis of B16/F10 cells by OT-I effectors primed with soluble OVA- or OCS-treated BMDCs. **(C and D)** Schematic and gating strategy for in vivo OT-I proliferation assays. **(E-H)** Analysis of cDC subsets: experimental workflow (E), gating strategy (F), identification of cDC1 and cDC2 populations (G), and P2 expression in sorted subsets (H). **(I and J)** CD69 expression on OT-I T cells after co-culture with OVA-pulsed cDC1s (I) or cDC2s (J). **(K-L)** P2 expression in BMDMs (K) and CD69 expression on OT-I T cells after co-culture with OVA-pulsed BMDMs (L). Data represent one of two independent experiments, and shown as mean ± SD; statistical significance was determined using an unpaired two-tailed Student's *t* test (A, I and J) and two-way ANOVA with Šídák's multiple comparisons test (B and L); ns, not significant (*P* > 0.05); ****P* < 0.001.


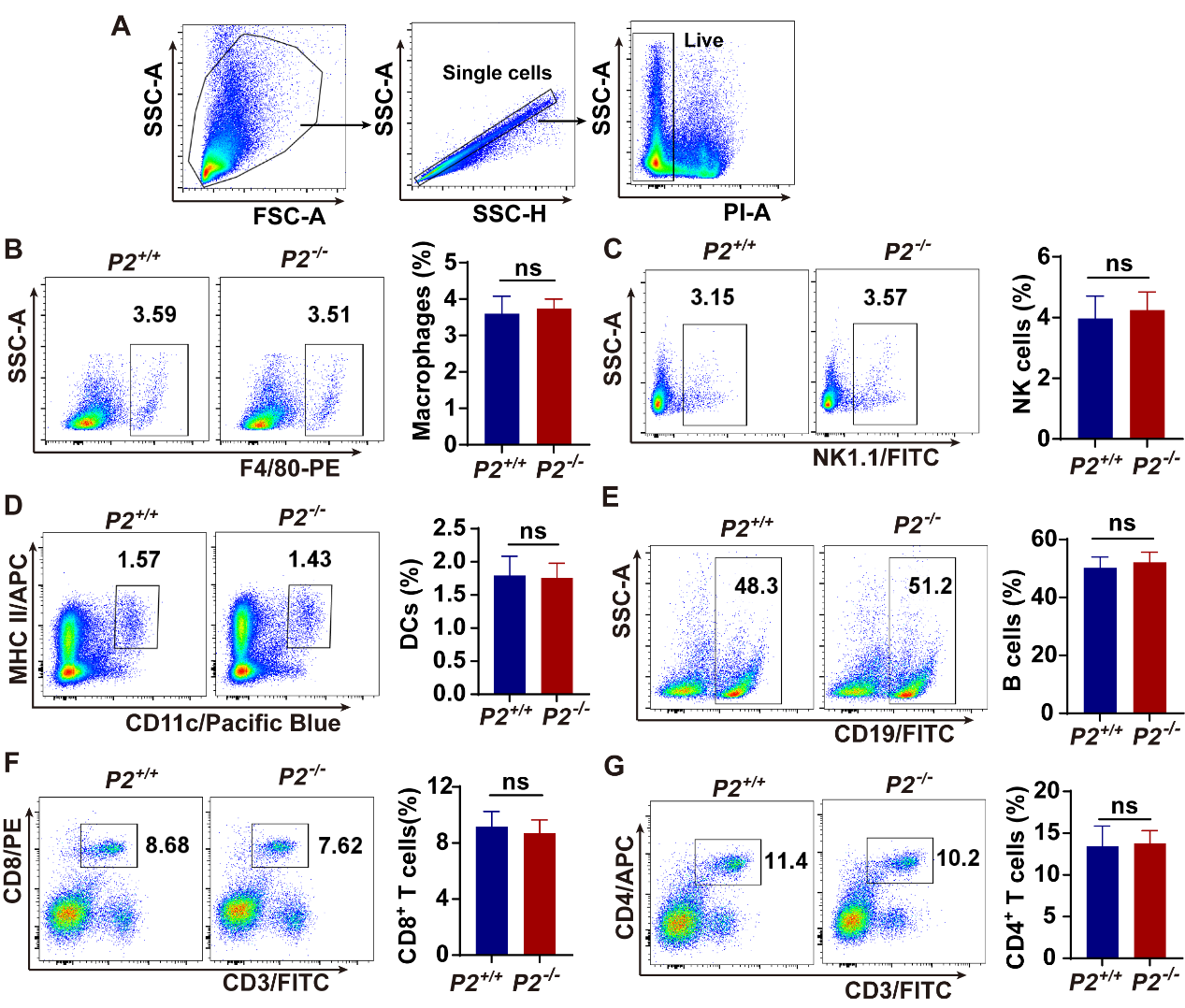


**Figure. S3.** P2 deletion does not affect the abundance of immune cells in spleens. **(A)** Representative gating strategy for flow cytometry. **(B)** Macrophages (F4/80^+^). **(C)** NK cells (NK1.1^+^). **(D)** Dendritic cells (CD11c^+^MHC II^+^). **(E)** B cells (CD19^+^). **(F)** CD8^+^ T cells (CD3^+^CD8^+^). **(G)** CD4^+^ T cells (CD3^+^CD4^+^). Data are presented as mean ± SD (n = 4); statistical analysis was performed using an unpaired two-tailed Student’s *t* test; ns, not significant (*P* > 0.05). All data are representative of three independent experiments.


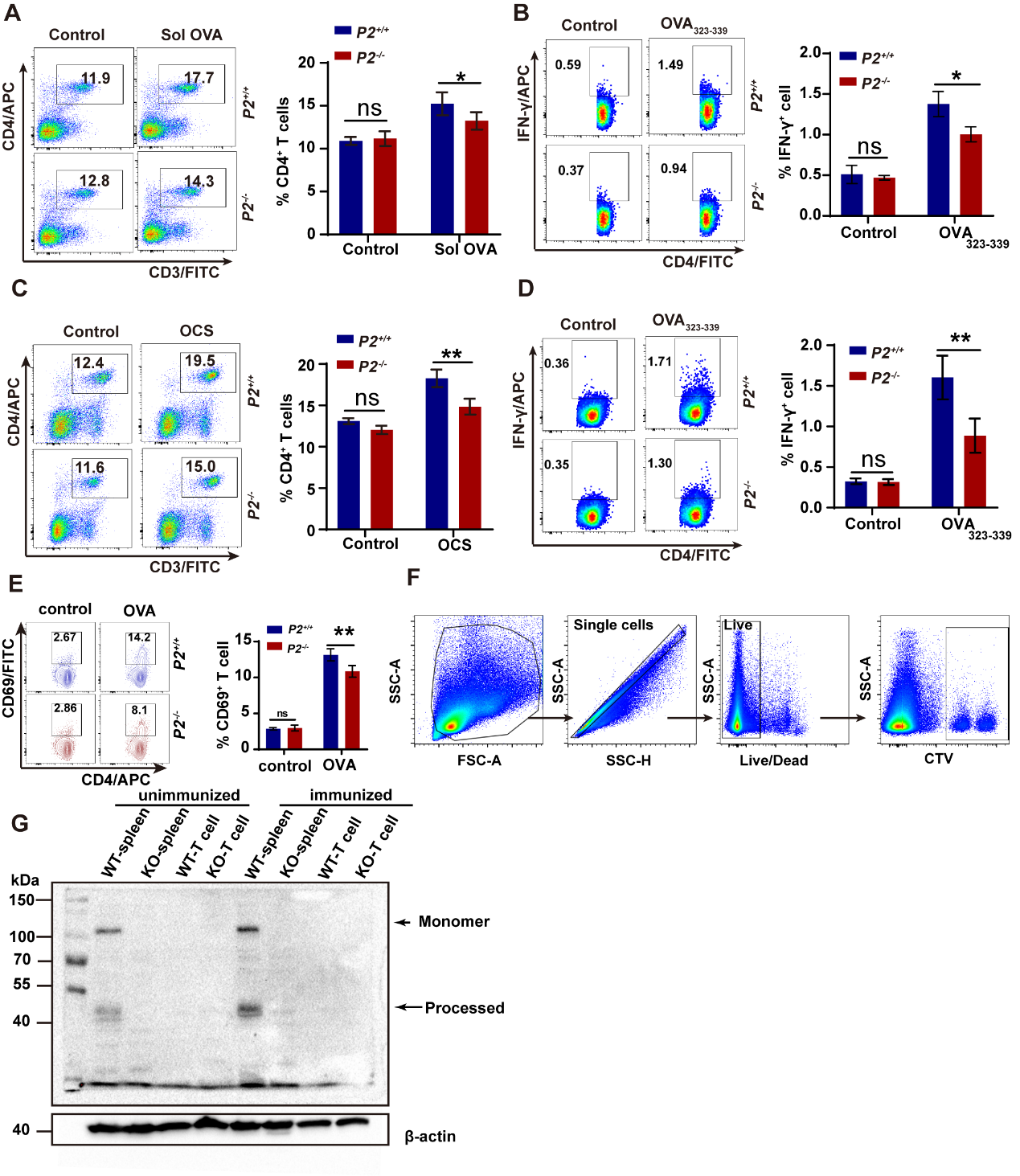


**Figure. S4.** P2 promotes endogenous CD4^+^ T cell responses. **(A and B)** Analysis of splenic CD4^+^ T cells in OVA-immunized mice showing total CD3^+^CD4^+^ T cell populations and IFN-γ-producing CD4^+^ T cells after OVA_323-339_ peptide restimulation. **(C and D)** Parallel analysis of splenic CD4^+^ T cells in OCSs-immunized mice demonstrating total CD3^+^CD4^+^ T cells and OVA-specific IFN-γ-producing CD4^+^ T cells. **(E)** Surface CD69 expression on OCSs-primed CD4^+^ T cells after co-culture with OVA-pulsed BMDCs. **(F)** Gating strategy for identifying CTV^high^ and CTV^low^ target cell populations. **(G)** Western blot analysis of P2 expression in splenic T cells from immunized mice. Data represent one of three independent experiments, and shown as mean ± SD; ns, not significant (*P* > 0.05), **P* < 0.05, ***P* < 0.01 by two-way ANOVA with Šídák's multiple comparisons test.


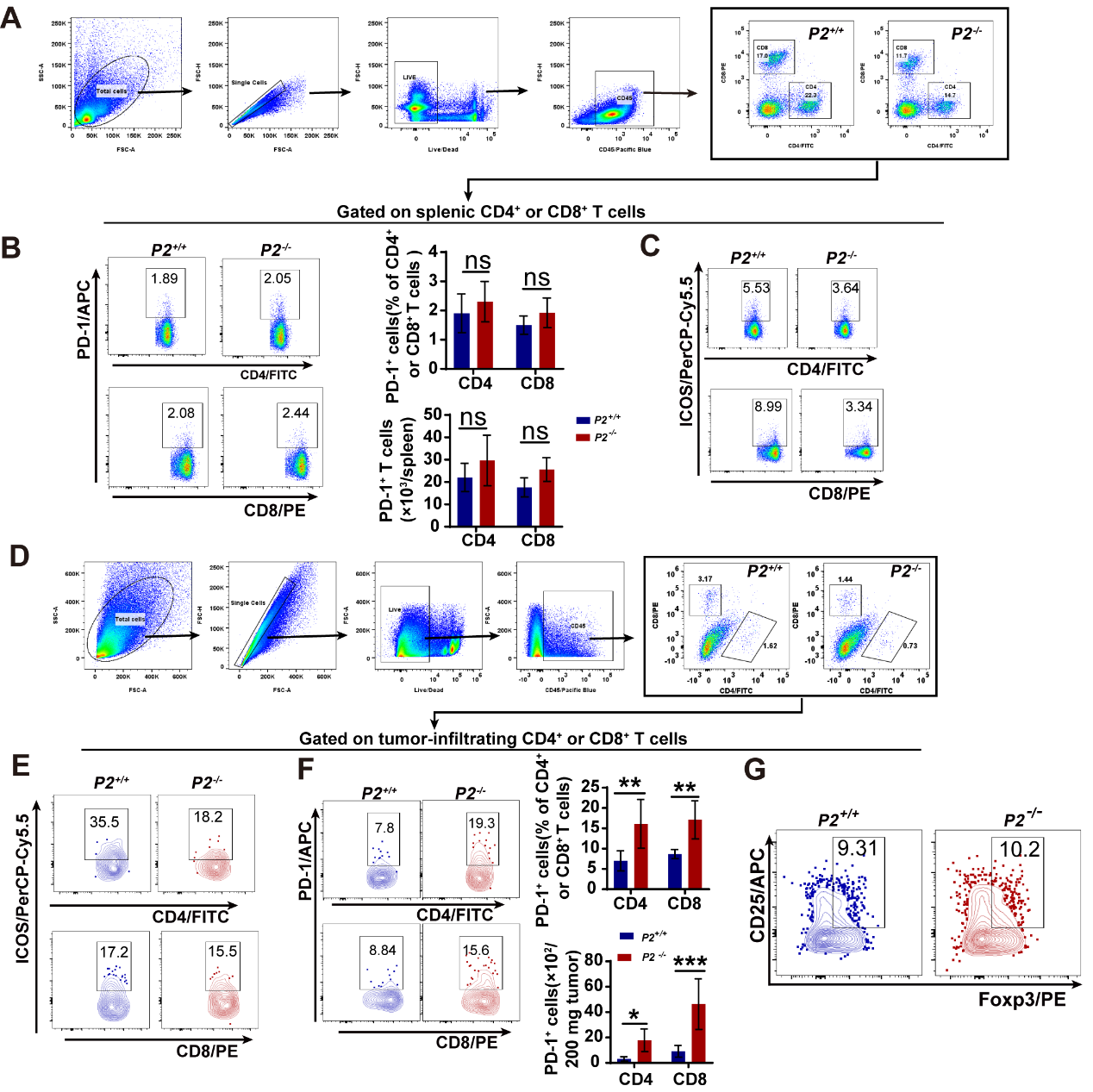


**Figure. S5.** P2 deficiency impairs T-cell infiltration and activation in the B16/F10-OVA tumor model. **(A-C)** Comparison of splenic T cells immune parameters in tumor-bearing mice. **(A)** Gating strategy for splenic CD8^+^ and CD4^+^ T cells. **(B and C)** Representative plots and corresponding quantification of PD-1 (B) and ICOS (C) expression on splenic CD8^+^ and CD4^+^ T cells. **(D-G)** Flow cytometric analysis of tumor-infiltrating CD8^+^ and CD4^+^ T cells from *P2^-/-^* and *P2^+/+^* mice. **(D)** Gating strategy for tumor-infiltrating CD8^+^ and CD4^+^ T cells. **(E)** Representative plots showing frequencies of tumor-infiltrating ICOS^+^CD8^+^ and ICOS^+^CD4^+^ T cells. **(F)** Representative flow cytometry plots showing PD-1^+^ cells on tumor-infiltrating T cells, with corresponding quantification on the right. **(G)** Representative plots showing frequencies of tumor-infiltrating Treg cells (CD4^+^CD25^+^Foxp3^+^). All flow cytometry plots without bar graphs represents representative data. Data are shown as mean ± SD and are representative of two independent experiments (n = 6 mice per group). Statistical significance was determined using two-way ANOVA with Šídák's multiple comparisons test (B and F); ns, not significant (*P* > 0.05); **P* < 0.05, ***P* < 0.01, ****P* < 0.001.


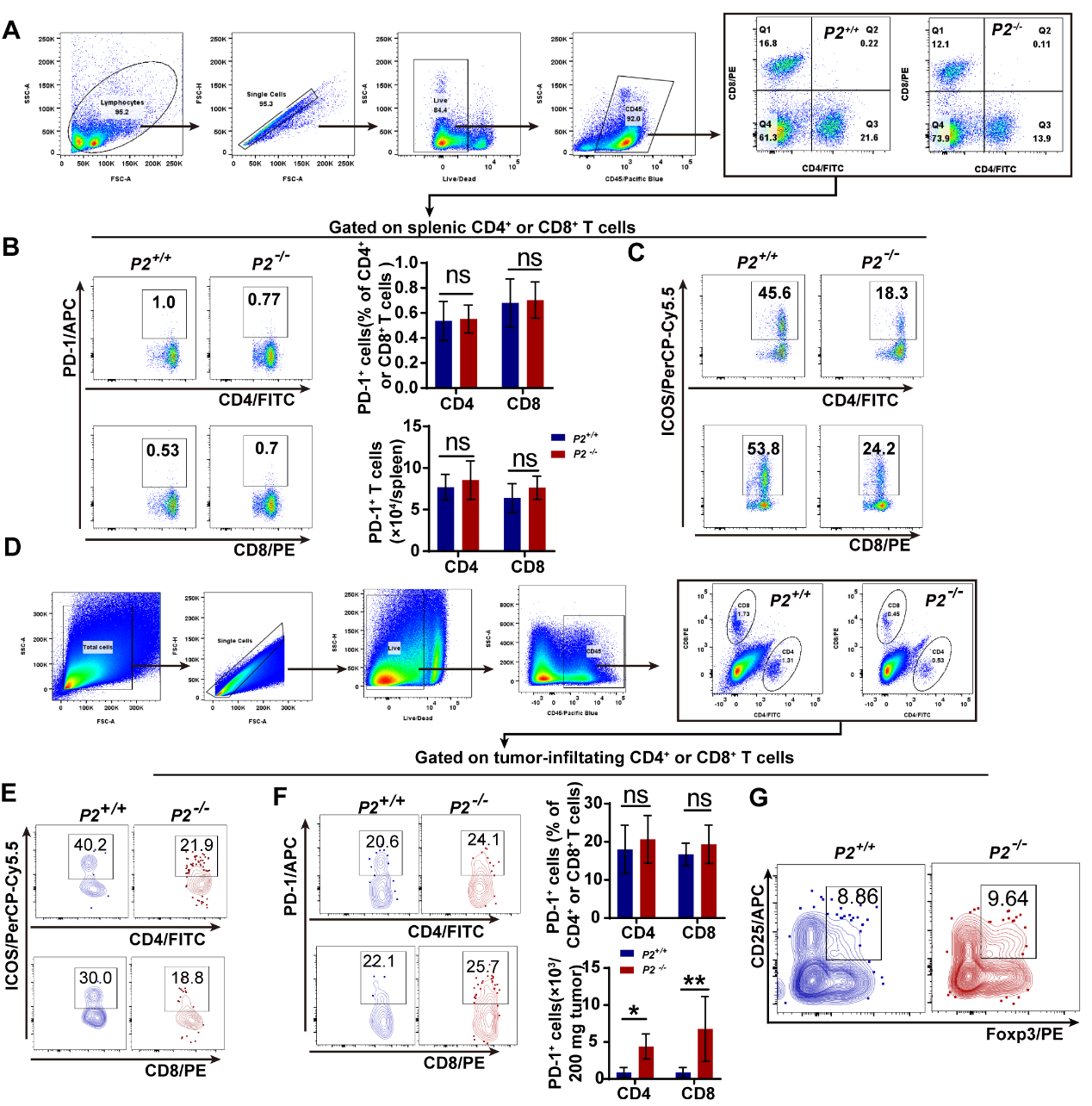


**Figure. S6.** P2 deficiency impairs T-cell infiltration and activation in the B16/F10 tumor model. **(A-C)** Comparison of splenic T cells immune parameters in tumor-bearing mice. **(A)** Gating strategy for splenic CD8^+^ and CD4^+^ T cells. **(B and C)** Representative plots and corresponding quantification of PD-1 (B) and ICOS (C) expression on splenic CD8^+^ and CD4^+^ T cells. **(D-G)** Flow cytometric analysis of tumor-infiltrating CD8^+^ and CD4^+^ T cells from *P2^-/-^* and *P2^+/+^* mice. **(D)** Gating strategy for tumor-infiltrating CD8^+^ and CD4^+^ T cells. **(E)** Representative plots showing frequencies of tumor-infiltrating ICOS^+^CD8^+^ and ICOS^+^CD4^+^ T cells. **(F)** Representative flow cytometry plots showing PD-1^+^ cells on tumor-infiltrating T cells, with corresponding quantification on the right. **(G)** Representative plots showing frequencies of tumor-infiltrating Treg cells (CD4^+^CD25^+^Foxp3^+^). All flow cytometry plots without bar graphs represents representative data. Data are shown as mean ± SD and are representative of two independent experiments (n = 6 mice per group). Statistical significance was determined using two-way ANOVA with Šídák's multiple comparisons test (B and F); ns, not significant (*P* > 0.05); **P* < 0.05, ***P* < 0.01.


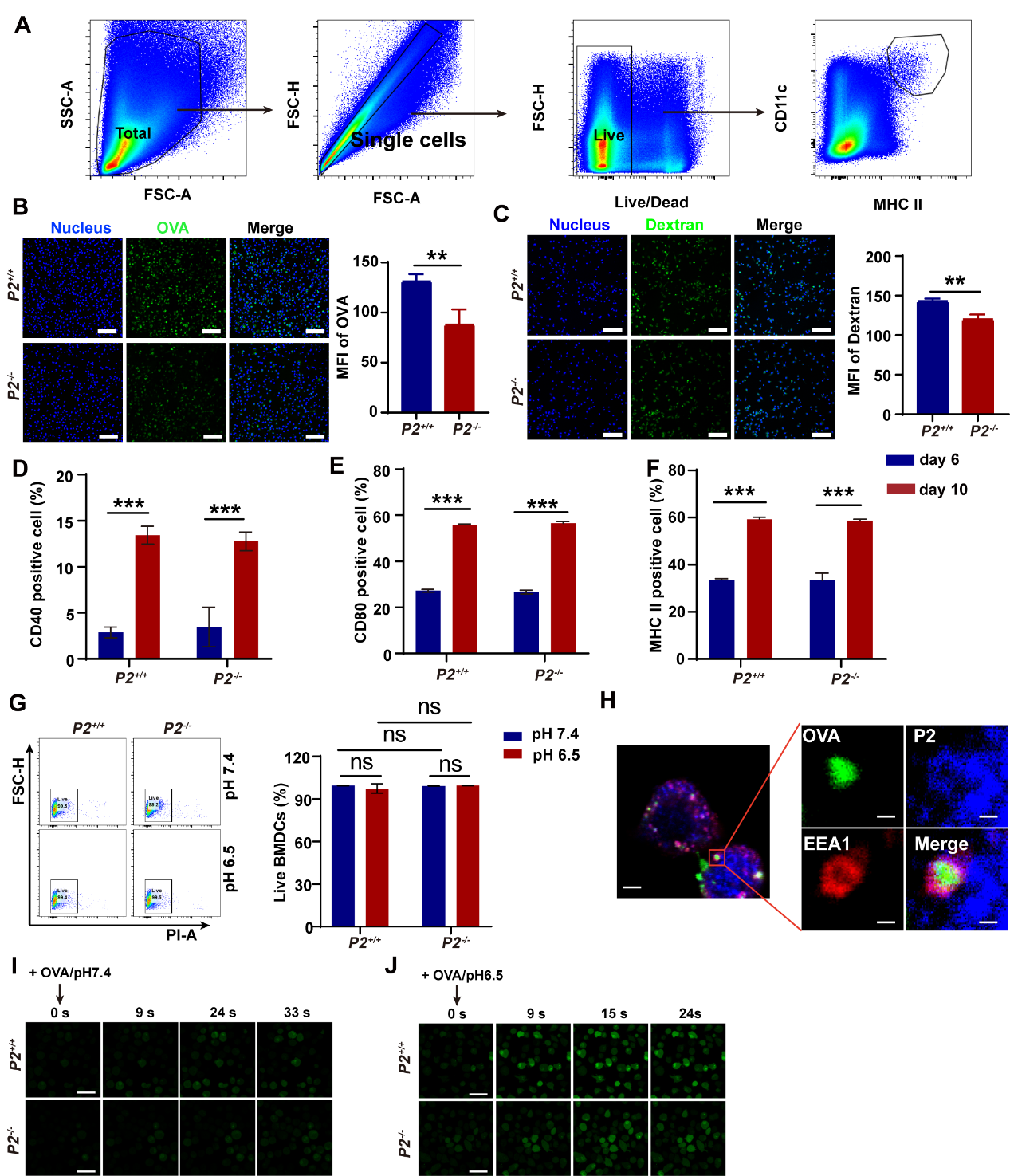


**Figure. S7.** P2 promotes antigen uptake. **(A)** Gating strategy for identifying CD11c^+^MHC II^+^ DCs. **(B and C)** Confocal microscopy analysis of FITC-OVA (B) and FITC-dextran (C) uptake by BMDCs showing intracellular MFI. Scale bar, 100 μm. **(D-F)** Quantitative comparison of CD40, CD80 and MHC II expression between day 6 and day 10 BMDCs. **(G)** pH-dependent OVA internalization and cell viability assessment using PI staining. **(H)** High-resolution imaging of OVA co-localization with P2 and EEA1 in early endosomes. Scale bars, 5 μm; zoomed-in images, 0.5 μm. **(I and J)** Time-course visualization of intracellular Ca²^+^ responses in BMDCs stimulated with OVA at pH 7.4 (I) or pH 6.5 (J). Scale bar, 20 μm. Data are shown as mean ± SD and are representative of two independent experiments (n = 3). Statistical significance was determined using an unpaired two-tailed Student’s *t* test (B and C) and two-way ANOVA with Šídák's multiple comparisons test (D-F and H); ns, not significant (*P* > 0.05); ****P* < 0.001.


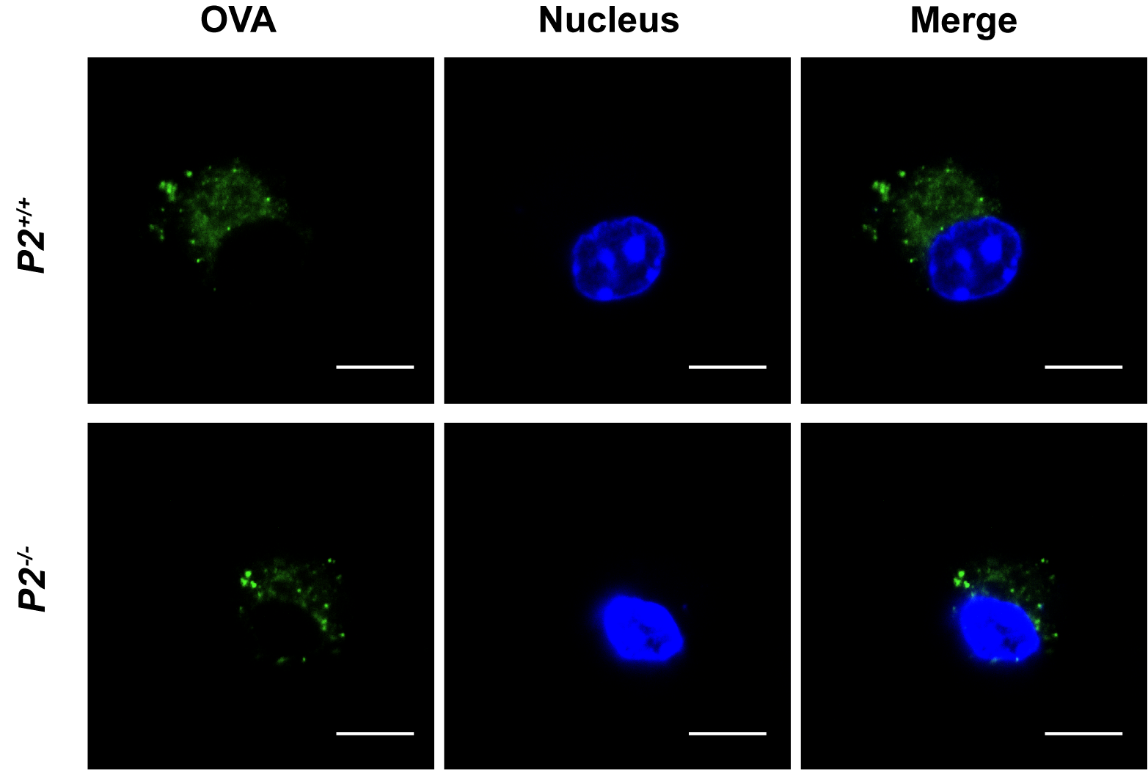


**Figure. S8.** *P2^-/-^* and *P2^+/+^* BMDCs were treated with 20 μg/ml FITC-OVA for 1 h, and the distribution of intracellular OVA was observed by confocal. Scale bar, 10 μm.


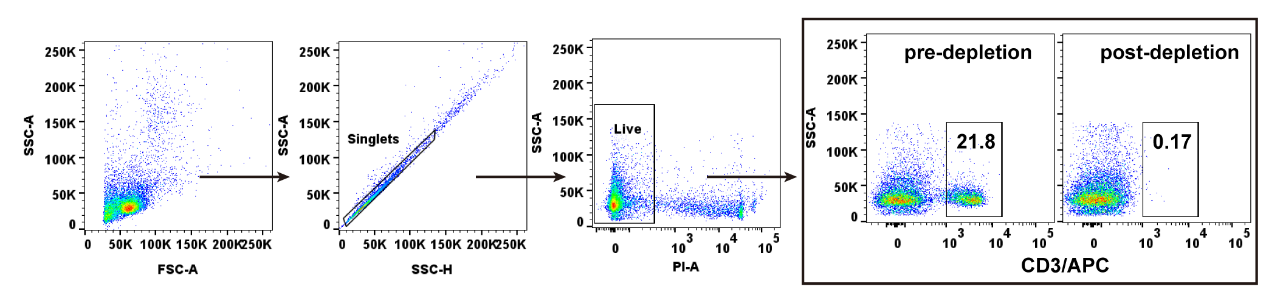


**Figure. S9.** T cells were removed from the splenocytes after positive sorting. The spleen of C57BL/6 mice was collected and prepared into cell suspension. The mouse CD3ε MicroBead Kit (Miltenyi Biotec) was used to remove total T cells from the splenocytes. Flow cytometry was used to examine the abundance of T cells before and after cell sorting.
